# Allelic Diversity of Acetyl Coenzyme A Carboxylase *accD*/*bccp* Genes Implicated in Nuclear-Cytoplasmic Conflict in the Wild and Domesticated Pea (*Pisum* sp.)

**DOI:** 10.3390/ijms20071773

**Published:** 2019-04-10

**Authors:** Eliška Nováková, Lenka Zablatzká, Jan Brus, Viktorie Nesrstová, Pavel Hanáček, Ruslan Kalendar, Fatima Cvrčková, Ľuboš Majeský, Petr Smýkal

**Affiliations:** 1 Department of Botany, Faculty of Sciences, Palacký University, 78371 Olomouc, Czech Republic; eli.novakova@seznam.cz (E.N.); zablatzka.lenka@seznam.cz (L.Z.); lubos.majesky@upol.cz (Ľ.M.); 2 Department of Geoinformatics, Faculty of Sciences, Palacký University, 78371 Olomouc, Czech Republic; jan.brus@upol.cz; 3 Department of Mathematical Analysis and Applications of Mathematics, Palacký University, 78371 Olomouc, Czech Republic; viktorie.nesrstova@gmail.com; 4 Department of Plant Biology, Faculty of Agronomy, Mendel University, 61300 Brno, Czech Republic; hanacek@mendelu.cz; 5 National Center for Biotechnology, Astana 010000, Kazakhstan; ruslan.kalendar@mail.ru; 6 Department of Agricultural Sciences, Viikki Plant Science Centre and Helsinki Sustainability Centre, University of Helsinki, FI-00014 Helsinki, Finland; 7 Department of Experimental Plant Biology, Faculty of Sciences, Charles University, 12844 Prague, Czech Republic; fatima.cvrckova@natur.cuni.cz

**Keywords:** acetyl-CoA carboxylase, hybrid incompatibility, hybrid necrosis, nuclear-cytoplasmic conflict, pea, reproductive isolation, speciation

## Abstract

Reproductive isolation is an important component of species differentiation. The plastid *accD* gene coding for the acetyl-CoA carboxylase subunit and the nuclear *bccp* gene coding for the biotin carboxyl carrier protein were identified as candidate genes governing nuclear-cytoplasmic incompatibility in peas. We examined the allelic diversity in a set of 195 geographically diverse samples of both cultivated (*Pisum* *sativum*, *P.* *abyssinicum*) and wild (*P.* *fulvum* and *P.* *elatius*) peas. Based on deduced protein sequences, we identified 34 *accD* and 31 *bccp* alleles that are partially geographically and genetically structured. The *accD* is highly variable due to insertions of tandem repeats. *P. fulvum* and *P. abyssinicum* have unique alleles and combinations of both genes. On the other hand, partial overlap was observed between *P.* *sativum* and *P.* *elatius*. Mapping of protein sequence polymorphisms to 3D structures revealed that most of the repeat and indel polymorphisms map to sequence regions that could not be modeled, consistent with this part of the protein being less constrained by requirements for precise folding than the enzymatically active domains. The results of this study are important not only from an evolutionary point of view but are also relevant for pea breeding when using more distant wild relatives.

## 1. Introduction

Reproductive isolation is an important component of species differentiation. Mechanisms that create reproductive barriers between once-conspecific organisms have long been a focus of evolutionary biology [1]. Although geographical separation plays a vital role in speciation [2], ecological factors also contribute [3]. Ecological selection favoring a particular cytoplasm has been described from various taxa [4,5]. Hybrid incompatibility due to the genetic divergence between the hybridizing parents has been theorized already by Bateson [6], Dobzhansky [7], and Muller [8]. Hybrid incompatibilities are proposed to be among the first genetic barriers to arise during speciation [9]. Although interspecific hybridization seems to be relatively frequent in plants, comparatively less is known about the reproductive barriers within species [10]. The most classical definition of the species relies on reproductive isolation, namely the inability to produce a viable offspring from inter-species hybridization [11,12]. Reproductive barriers might be broadly classified into prezygotic (pre-pollination) and postzygotic (post-pollination) ones [13]. Pre-pollination isolation mechanisms, such as habitat divergence, temporal isolation, pollinator isolation, and mating system divergence, are usually more effective than post-pollination isolation [2]. 

Interactions among nuclear-encoded genes can lead to diverse forms of hybrid incompatibility via multiple gametophytic and sporophytic mechanisms [9]. The identification of so-called ‘speciation genes’ is of interest because their knowledge would offer clues to the ecological settings, evolutionary forces, and molecular mechanisms that drive the divergence of populations and species [12,14]. A speciation gene can be strictly defined as a gene that contributes to the splitting of two lineages by reducing the amount of gene flow between them [12].

Until recently, characterization of genetic incompatibility has largely focused on the differences between species and on nuclear incompatibilities [2,12,15,16,17]. As a result, the importance of cytonuclear incompatibility (i.e., incompatibility between the nuclear and organelle genomes) in driving the early stages of speciation received less attention [10]. There has been long co-evolution between the nuclei and organelles. Molecular data indicate a large degree of interdependence between the cellular sub-genomes [18]. The subdivided eukaryotic genome has resulted from a massive restructuring and intermixing of the genomes of the initially free-living symbiotic partner cells with loss, intracellular transfer, and gain of genetic information, with resulting high interdependence and mutual “fine tuning” of both genomes that can easily become disrupted upon intraspecific hybridization. Cytonuclear incompatibilities are predisposed to be substantial contributors to reproductive isolation and speciation [19,20]. Empirical studies have shown that intrinsic postzygotic barriers to reproduction—hybrid inviability and hybrid sterility—evolve through mechanisms consistent with the classic Bateson–Dobzhansky–Muller model [9]. As adaptive or nearly neutral substitutions accumulate in diverging lineages, these may in a particular lineage become fixed in a state incompatible with that in the other lineage. As a result, the hybrid dysfunction occurs when such incompatible alleles are brought together. The genetic basis for hybrid sterility has been studied in several plants, such as *Solanum* [21], *Oryza* [22], *Mimulus* [23], *Oenothera* [24], *Arabidopsis lyrata* [25], and *A. thaliana* [26,27]. There are two classes of cytonuclear hybrid incompatibility: cytoplasmic male sterility (CMS), due to mitochondrial-nuclear mismatch, and cytonuclear chlorosis, caused by plastome–nuclear incompatibilities. Organelle genomes have a reduced population size and lack sexual recombination [28]. These characteristics both increase genetic drift, and lead to potential accumulation of deleterious mutations and selection for compensatory evolution in interacting nuclear genes. Due to these factors, cytonuclear incompatibilities have been proposed to be among the first genetic incompatibilities to arise, influencing the earliest stages of speciation [10,19,20,24,29]. 

Most known plant sterility loci have been found in the mitochondrial genome, causing CMS characterized by the absence of viable pollen. The genetics of hybrid CMS are remarkably conserved across flowering plants. Molecular genetic studies indicate that CMS typically results from rearrangements in the mitochondrial genome [30,31]. As mitochondria are usually maternally inherited, CMS is typically transmitted through the ovules. In contrast, nuclear genes are transmitted through both ovules and pollen. This difference in inheritance patterns creates a genetic conflict between nuclear and cytoplasmic genes. Hybrid nucleo-organelle dysfunction can result in post-zygotic hybridization barriers that usually manifest as differences in the offspring of reciprocal crosses owing to non-Mendelian inheritance of organelles. Asymmetry in reproductive isolation appears to be common and taxonomically widespread among plant species. Plastids can also contribute to nucleo-cytoplasmic incompatibility. Although cytonuclear chlorosis or albinism of hybrids is not as common as CMS, these have been widely observed, and their implications for speciation were recognized early on [32,33,34,35]. The role of plastids in speciation processes is known from species with a biparental mode of plastid inheritance, e.g., *Geranium, Pelargonium* and *Medicago* [36], and mainly from genus *Oenothera*, which became one of the models for studying plant evolution [24]. Various incompatible phenotypes have also been reported from *Rhododendron, Hypericum, Trifolium, Zantedeschia*, and *Pisum* [24]. Cyto-nuclear co-adaptation has been described in *Arabidopsis thaliana* [18] and demonstrated to affect its adaptive traits [37]. Interestingly, crop domestication may also increase the likelihood that genes causing incompatibility become fixed in the population through genetic hitchhiking [38].

The plastid *accD* gene coding for the acetyl-CoA carboxylase beta subunit and the nuclear gene *bccp* coding for the biotin carboxyl carrier protein of acetyl-CoA carboxylase were nominated as candidate genes responsible for nuclear-cytoplasmic incompatibility in peas based on data from crosses between wild and domesticated pea forms [39]. Incompatible hybrids exhibit chlorophyll deficiency, reduction of leaf size low pollen fertility, low seed set, and poorly developed roots [40]. The acetyl-CoA carboxylase (ACCase) complex is involved in the biosynthesis of fatty acids, which takes place in the plastids [40]. ACCase belongs to a group of biotin dependent carboxylases, catalyzing acetyl-coenzyme A carboxylation to malonyl coenzyme A and providing the only entry point for all carbon atoms in the fatty acid synthesis pathway [41]. Uniquely in Eukaryota, plants have two distinct ACCases: one eukaryotic-like homomeric multidomain ACCase in the cytosol and a bacterial-like heteromeric ACCase within the plastids [41]. The heteromeric form of ACCase is found in prokaryotes and the plastids of Viridiplantae. Presumably, all genes encoding ACCase subunits initially resided in the plastid genome after the original endosymbiotic event in algae and underwent sequential transfer to the nuclear genome [42]. Plastid ACCase participates in fatty acid synthesis, whereas the cytosolic enzyme is engaged in the synthesis of very long chain fatty acids, phytoalexins, flavonoids, and anthocyanins. Plastid-localized ACCD enzyme is responsible for catalyzing the initial tightly-regulated and rate-limiting step in fatty acid biosynthesis. Nuclear encoded Biotin Carboxyl Carrier Protein (BCCP) is a part of the enzyme Acetyl-CoA carboxylase complex and serves as a carrier protein for biotin and carboxybiotin throughout the ATP-dependent carboxylation of acetyl-CoA to form malonyl-CoA. The resulting Acetyl-CoA carboxylase is a heterohexamer composed of the biotin carboxyl carrier protein, biotin carboxylase, and two subunits each of the ACCase subunit alpha and the ACCase plastid-coded subunit beta [40].

The plastid ACCase of legumes (Papilionoideae) consists of four subunits, each coded by a separate gene: biotin carboxylase (*accC*), biotin carboxyl carrier protein (*accB=bccp*), alpha-carboxyltransferase (*accA*), and beta-carboxyltransferase (*accD*). The genes coding *accC, accB,* and *accA* are localized in the nuclear genome, whereas the *accD* gene is localized in the plastid genome [42]. Multiple independent lineages have experienced accelerated rates of substitution in similar subsets of non-photosynthetic genes, including *accD* (in legumes [43,44,45] and in Oleaceae [46]). In *Silene* (Caryophyllaceae) species with accelerated plastid genome evolution, the nuclear-encoded subunits of the ACCase complexes are also evolving rapidly, indicating a strong positive selection [47]. Such patterns of molecular evolution in these plastid–nuclear complexes are unusual for ancient conserved enzymes but resemble cases of antagonistic coevolution between pathogens and host immune genes. Genetic characterization of hybrid necrosis in crosses between tomato species [48] and between *Arabidopsis* ecotypes [49,50] has revealed that incompatibilities among complementary disease resistance genes might play such a role in the evolution of hybrid inviability [51].

In this work, we explored the allelic diversity of *accD*/*bccp* in the geographically diverse set of wild pea (*Pisum* sp.). The *accD*/*bccp* are recently identified genes underlying nuclear-cytoplasmic incompatibility in *Pisum* sp. [39]. We sought to map the allelic combinations of *accD*/*bccp* occurring in nature to determine geographic patterns in their distribution, and to identify possible relationships to pea genetic diversity.

## 2. Results

### 2.1. Structure and Variation of accD Gene

The *accD* gene is located between positions 70,882 and 72,654 in the *P. sativum* cv. Feltham First (HM029370) reference chloroplast genome, resulting in a 1772 bp DNA encoding a protein of 432 amino acid residues. The primers used in our study were designed to match the most conserved region and were located close to the ends of the *accD* coding sequence. Consequently, we did not capture the very 5′ and 3′ end of the coding sequence due to quality trimming. The beginning and end of the *accD* sequence, comprising 48 nt from the start codon and 58 nt from the stop codon, consequently missing the first 16 and last 19 codons, were thus excluded from the subsequent analysis.

**Figure 1:**
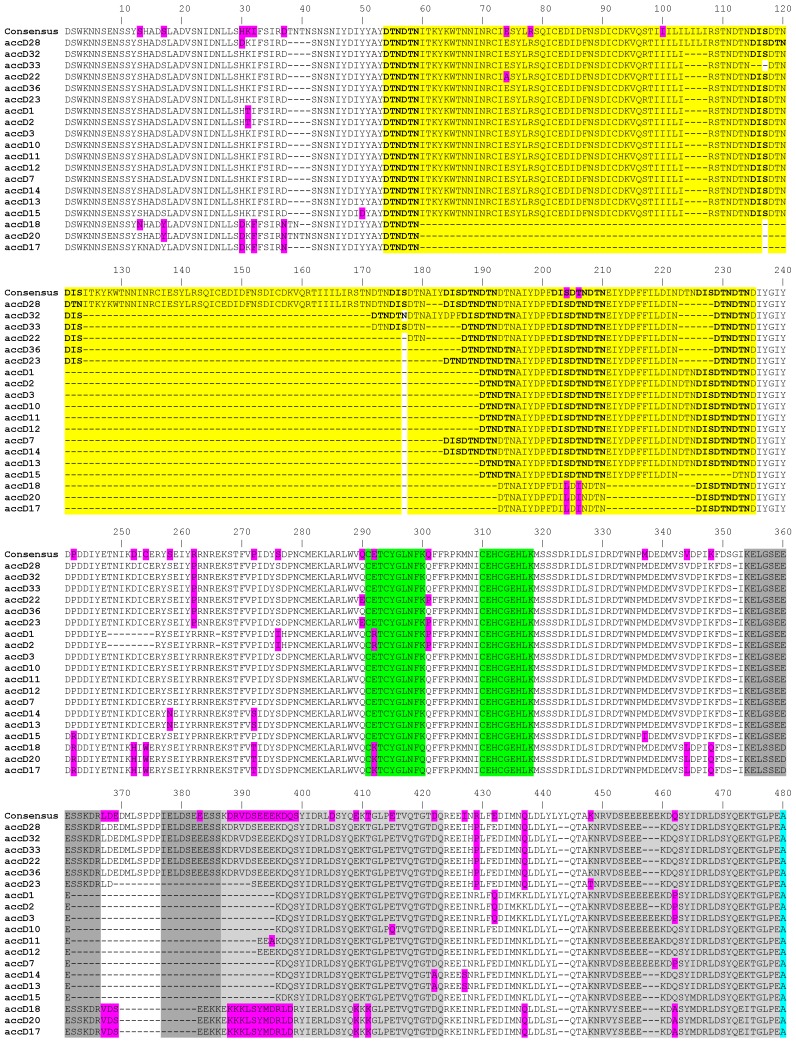
The alignment of amino acid sequences of all identified *accD* alleles. The figure only shows the region from 1 to 480 amino acid residues. The colored regions show the 5 translated repeats, polymorphic amino acid exchanges (in magenta), Zn-finger (boxed), acetyl-CoA binding (light blue), coA carboxylation catalytic (dark blue), and carboxybiotin binding (in green) sites. Residues in purple are point mutations in at least one haplotype. There are no indels after position 480 (for full see Figure S1).

The length of the *accD* gene within our studied material ranged from 1403 bp to 1859 bp at DNA level and from 467 to 619 amino acid residues, respectively (GenBank accession numbers MK619486—MK619678). In the studied set of 195 accessions, there was extraordinary variation in the gene length, due to the occurrence of 13 indels whose length varied between 3 and 167 nucleotides. This variation is due to insertions consisting of tandem repeats of 10-150 bp units present in 1 to 37 nearly identical copies, all in the same (i.e., direct) orientation relative to each other (Figure 1). The repetitive sequences can be divided into 6 categories. In the shortest 1403 bp allele (JI1010, *P. fulvum*) there are four, three, and one repeats of 9 to 12 bp long. These expand in the longest 1859 bp allele (JI267, *P. elatius*), which has 37 repeats of 10 to 33 bp, 1 repeat of 57 bp, 1 repeat of 102 bp, and 1 repeat of 149 bp. We identified the main five longest tandem repeats blocks, which consist of two or three individual blocks of different lengths and degrees of identity. These blocks are not identical and contain many nucleotide changes and triplet duplications. Such repeats were identified by the presence of small, almost identical blocks, that are part of larger tandem repeats. The first tandem repeat block is the most complex and most degenerate, consisting of three sequential blocks (highlighted in yellow in Figure 1, Figure S1). These blocks are of different lengths and are degenerate to varying degrees from each other. The most similar are first two blocks (89%), which differ by 3 amino acids and by the insertion L-I-L-I for a total of 64 amino acid residues. Characteristic for this tandem repeat is the presence of multiple duplications of three amino acids D-T-N alone or together with D-I-S. The complex, degenerate, and mixed tandem repeat is also the penultimate (3 and 4 grey blocks). This tandem repeat has multiple duplications of five amino acid stretch of S-E-E-E-K. The remaining repeats consist of two blocks separated from each other by 7 or 9 amino acids (Figure 1).

### 2.2. Variation in Nuclear bccp Gene

The predicted ORF of the *bccp* gene encoding the biotin carboxyl carrier protein of *P. sativum* cv. Cameor from the pea RNA atlas is 873 bp long and encodes a protein of 290 amino acids. In the pea RNA atlas, this is represented by the ubiquitously expressed PsCam051640 transcript, which corresponds to Tayeh et al. (2015) map PsCam051640 at LGIII. The genomic DNA extracted from the shotgun genome sequence is 5906 bp, with 9 exons interspersed by 8 introns (Exon 1 is 234 bp, exon 2 is 206 bp, exon 3 is 76 bp, exon 4 is 54 bp, exon 5 is 262 bp, exon 6 is 62 bp, exon 7 is 69 bp, exon 8 is 46, and exon 9 is 265 bp). The respective introns are 1170, 541, 263, 874, 111, 856, 84, and 733 bp. The following analysis was conducted on cDNA, avoiding introns. The detected polymorphism, thus, only concerns the coding sequence, and is correspondingly lower than that expected for the complete locus. Notably, to obtain sufficient PCR product we had to perform out two consecutive nested PCR amplifications. This likely reflects the relatively low expression level of the gene in young leaf tissue. There were altogether 39 variable positions and no indels in a total of 195 studied accessions (NCBI accession numbers MK644626—MK644819). These identified 31 protein *bccp* variants (Table S1). Sixteen analyzed *P. fulvum* accessions had three *bccp* alleles (*bccp*1/2/3) separated by 4 to 10 amino acid changes from the nearest *P. elatius* alleles. From domesticated *P. sativum* landraces (60 acc.), 16 had the *bccp*_22, and six had the *bccp*_18 allele. From the independently domesticated Ethiopian pea *P. abyssinicum* (24 acc.), 19 had the specific *bccp*_26 allele, shared with two *P. elatius* accessions (PI343978, PI343979 from Turkey), four had the *bccp*_20 allele, separated by one or two amino acid exchanges from nearest *P. elatius*. Ninety-five analyzed *P. elatius* accessions had the largest diversity (all together 28 distinct *bccp* alleles, Table S1).

### 2.3. Network and Maximum Parsimony Analyses

**Figure 2:**
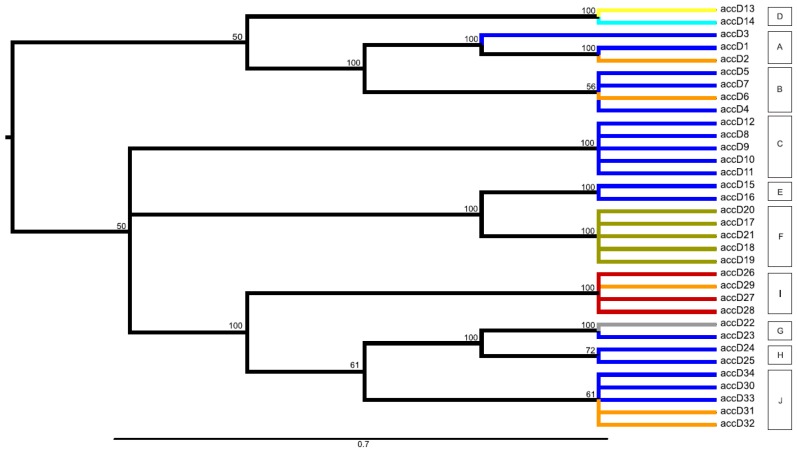
Midpoint-rooted consensus tree for the *accD* gene presenting the most parsimonious relationships among the identified 34 alleles within the studied world-wide pea collection. The consensus tree is build up from the 18 equally parsimonious trees (length 73, consistency index 0.900; retention index 0.972; composite index 0.892). Branch coloring follows the species presence of particular alleles: olive green = alleles observed only within *P. fulvum*; grey = alleles shared among *P. fulvum* and *P. elatius*; orange = alleles shared among *P. sativum* and *P. elatius*; red = alleles observed only for *P. sativum*; turquoise = alleles shared among *P. abyssinicum* and *P. sativum*; yellow = alleles observed only within *P. abyssinicum*; blue = alleles observed only within *P. elatius*. Bootstrap support ≥ 50 is shown above branches.

**Figure 3:**
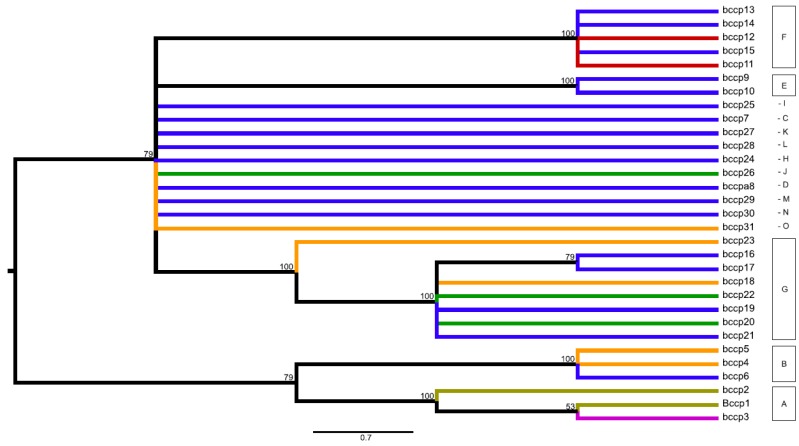
Midpoint-rooted consensus tree for the *bccp* gene presenting parsimonious relationships among the identified 31 alleles within the studied world-wide pea collection. The consensus tree built from the 19 equally parsimonious trees (length 42, consistency index 0.762; retention index 0.900; composite index 0.793). Branch coloring follows species presence of particular alleles: olive green = alleles observed only within *P. fulvum*; magenta = alleles shared among *P. fulvum* and *P. sativum*; orange = alleles shared among *P. sativum* and *P. elatius*; red = alleles observed only for *P. sativum*; green = alleles shared among *P. abyssinicum*, *P. sativum* and *P. elatius*; blue = alleles observed only within *P. elatius*. Bootstrap support ≥ 50 is shown above branches.

Various approaches in the visualization of the data through networks and maximum parsimony (MP) analysis produced a very similar view, with only minimal differences. For further interpretation of clustering of identified alleles into larger groups, the consensus maximum parsimony tree method was used. This produced a very similar clustering of alleles as inspected networks (Median network, NeighborNet, SplitDecomposition networks; not shown). The MP analysis found 18 equally parsimonious trees for the *accD* gene (length 73 steps) and 19 for the *bccp* (42 steps) (Figure 2, Figure 3). The resulting trees contained several polytomies. This is because of a large part of the total sequence variability being due to indels in the case of *accD*, and this information was not included into the MP analysis. In addition, a number of homoplasious mutations were also excluded, with the resulting trees contained several polytomies. However, as we were not interested in the assessment of the gene phylogeny, we did not try to interpret these polytomies. Produced clades (with a rather high bootstrap support) were very similar to groups inferred from the network analyses. Based on the similarity in the grouping of alleles between inspected networks and the MP analysis, the groups of alleles were inferred from the consensus MP tree for both investigated genes. For the *accD* gene 10 groups (A–J) were inferred; 15 groups were inferred for the *bccp* gene (A–O) were inferred (Figure 2, Figure 3, Table S1). The *accD* gene group D (comprising alleles *accD*_13 and *accD*_14) was specific for *P. abyssinicum*, except for one sample of *P. sativum* from Montenegro (accession n° PI357292), which also possessed the *accD*_14 allele. Group F (comprising alleles **accD**_17/18/19/20/21) was specific for *P. fulvum*. Accessions of *P. elatius* and landraces of *P. sativum* were represented by multiple alleles belonging to different groups. 

In the case of the *bccp* gene, *P. abyssinicum* was represented by groups J (allele *bccp*_26) and G (alleles *bccp*_20/22). However, in contrast to the *accD* gene, inferred alleles were not specific for *P. abyssinicum*, but were also found within *P. elatius* and samples of *P. sativum* (Figure 3, Table S1). The three identified alleles observed for *P. fulvum* (*bccp*_1/2/3) clustered together and represented group A. Two of these alleles were specific (*bccp*_1/2) for *P. fulvum* and one (*bccp*_3) was shared with two samples of *P. sativum* from Greece (JI1525 and JI2573). The identified alleles for the investigated accessions of *P. elatius* fall within 12 groups and for *P. sativum* within six groups, which were shared between these two species (Table S1). 

### 2.4. Frequency of Amino Acid Substitutions and Their Distribution

Analysis of the nuclear encoded *bccp* gene in a panel of 179 samples of 809 sites resulting in 269 analyzed codons revealed 196 synonymous sites (Pi(s): 0,00616 Pi(s), Jukes & Cantor: 0,00620) and 610 non-synonymous sites (Pi(a): 0,00553, Pi(a), Jukes & Cantor: 0,00556). This resulted in a Ka/Ks ratio of 0.895. Despite the presence of frequent insertions and deletions, the *accD* sequence could be translated into protein. The analysis covered 1306 sites (e.g., 425 codons). Nucleotide diversity analysis of *accD* showed 278 synonymous sites (Pi(s): 0,00390, Pi(s), Jukes & Cantor: 0,00391) and 997 non-synonymous sites (Pi(a): 0,01048, Pi(a), Jukes & Cantor: 0,01064). This resulted in a high Ka/Ks ratio of 2.726, which indicates positive selections and accelerated evolutions.

Analysis of protein sequence revealed that the ACCD protein has a ClpP protease/crotonase domain (IPRO 29045; region of 251 to 296 and 384 to 584 amino acids), coiled coil domain (region of 380 to 407 amino acids), an acetyl-CoA-carboxyltransferase N terminal domain (IPRO 11762; in region of 226 to 590 amino acids), and a zinc finger (230–252 amino acids) domain (Figure 1). The BCCP protein has a biotin/lipoyl attachment (IPRO 000089) domain (region of 207 to 280 amino acids) and a carboxytransferase (CT) interaction site (239G-284F-249G-250A-257D), where 249G is a conserved biotinylation site.

**Figure 4:**
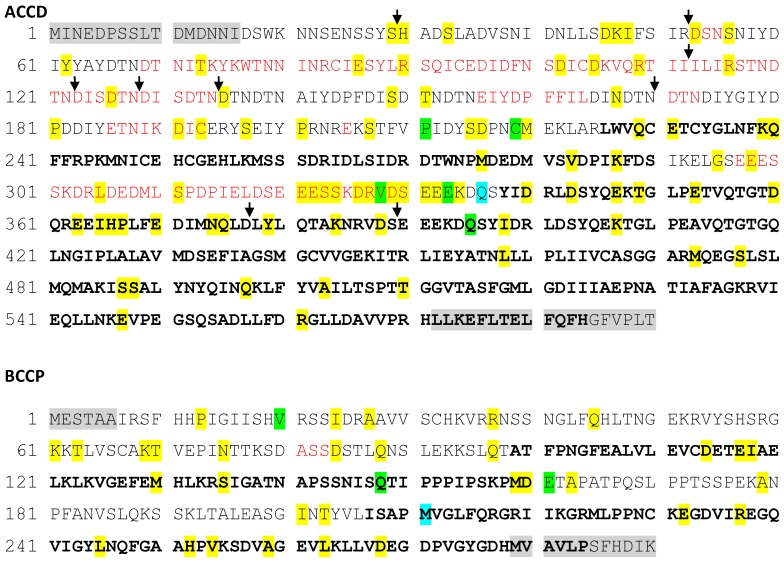
Parts of the ACCD (**A**) and BCCP (**B**) protein sequences covered by the molecular model are marked in bold. Residues on gray background were not covered by the population sequence alignment. Residues exhibiting one, two, or more allelic variants are shown on a colored background. Residues shown in red are deleted only in some alleles. Black arrows indicate the location of insertions in some alleles.

We next attempted to investigate the location of the individual amino acid substitutions, and the conspicuous indels found in *accD*. This was performed with respect to the 3D folding of both ACCD and BCCP proteins, to the extent that we were able to predict their spatial structure by threading on experimentally characterized related templates. We could produce only partial models for both proteins (File S1, S2 For ACCD, the model covered approximately 43% of the sequence, corresponding to the C-terminal portion of the protein). The N-terminal region and an additional loop within the modelled segment were disordered in the prediction. For the BCCP protein, approximately 45% of the sequence was covered by the best templates but only two short separate fragments from this domain could be reliably modeled; the rest of the molecule was disordered in the prediction (Figure 4, Table 1).

Remarkably, mapping of the identified protein sequence polymorphisms revealed that most of the above-described repeat and indel polymorphisms in the ACCD sequence map to sequence regions could not be modelled due to the lack of suitable templates and intrinsic disorder. This is consistent with this part of the protein being less constrained by requirements for precise folding than the enzymatically active domain. Point mutations were also somewhat enriched in the part of the ACCD protein that was not modeled. However, no such bias was detected for BCCP (Figure 4, Table 1).

### 2.5. Allelic accD/bccp Combinations

**Figure 5:**
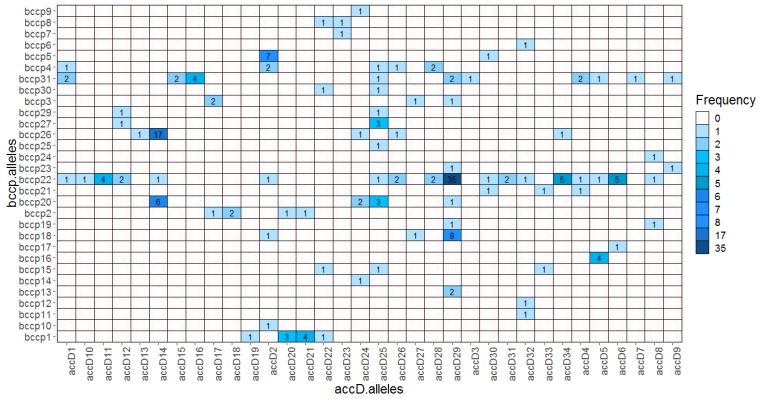
Heatmap of identified pairwise ACCD/BCCP allelic combinations.

We found 34 *accD* and 31 *bccp* alleles yielding altogether 1054 possible combinations. Within the wild pea (*P. elatius*) we detected 61 combinations (Table S2). Most of these combinations (45) were found only once. Cultivated *P. sativum* landraces had 20 combinations; the most frequent were *accD*_29/*bccp*_22 (30), followed by *accD*_29/*bccp*_18 (8). *P. abyssinicum* accessions had 4 distinct combinations, with *accD*_14/*bccp*_26 being predominant (17). *P. fulvum* had 9 combinations, *accD*_21/*bccp*_1 (4), *accD*_20/*bccp*_1 (3), and *accD*_17/*bccp*_3 (2). The only exception in our *P. fulvum* set was JI2539 from Israel, which had *accD*_22 (*accD*_G lineage) shared with *P. elatius*. There were two *bccp* alleles (*bccp*_22 and *bccp*_31) that formed the highest number of combinations with 18 and 10 *accD* alleles, respectively. Conversely, two *accD* alleles, *accD*_29, *accD*_25, and *bccp*_22, *bccp*_31 formed 8, 9, and 19, 10 combinations, respectively. Notably, the most frequent combination found in *P. sativum* landraces *accD*_29/*bccp*_22 was found in these high occurrence alleles (Figure 5).

### 2.6. Relationship to Pisum Genetic Diversity

Having previously analyzed genetic diversity based on genome-wide sampled polymorphism [52,53], we examined the distribution of both *accD* and *bccp* alleles within respective genetic groups. Cultivated *Pisum sativum* accessions can be divided into two (nr. 3 and 6) equally abundant (24 and, 27 accessions, respectively) groups. The independently domesticated Ethiopian pea (*P. abyssinicum*) forms a separate (nr. 7) group (Table S1). With respect to *accD/bccp* alleles, *accD*_29 and *bccp*_22 alleles predominate in 60 analyzed *P. sativum* accessions (41, 38 accessions respectively) (Supplementary Table S1), while all 24 *P. abyssinicum* accessions had single unique *accD*_14 and *bccp*_26 (17 acc.), *bccp*_20 (5 acc.) and *bccp*_22 (JI1974) alleles corresponding to its separate domestication history and associated bottleneck. *P. fulvum* as a separate species forms a separate genetic group (nr. 2) and has also distinct and the most distant *accD* (*accD*_17-21) and *bccp* (*bccp*_1-3) alleles, separated by 39 to 40, and 7 to 8 amino acids, respectively, from the closest *P. elatius* alleles. On the contrary, wild *P. elatius* is genetically the most diverse and has seven genetic groups (Trněný et al. 2018), one of which (nr. 3) overlaps with *P. sativum*. This diversity is also reflected with 22 different *accD* and 25 *bccp* alleles, respectively. The most abundant are *accD*_25 (13 acc.), *accD*_29 (9 acc.), *accD*_2 (11 acc.), and *bccp*_22 (28 acc.), and *bccp*_31 (16 acc.) (Table S1). There is only a partial relationship between the genome wide DARTseq and *accD*/*bccp* based diversity. Genetic group nr. 10 of *P. elatius* accessions from the Caucasus region has the most distinct *accD*_30, 31, 34, but not *bccp* alleles. Similarly, genetic groups nr. 4 and 5 have a high proportion of *accD*_2 (8 acc.) and *accD*_15/16 (5 acc.) alleles in samples from Israel or eastern Turkey and Georgia, respectively. No clear genetic group assignment was found for *bccp* alleles within *P. elatius* accessions.

### 2.7. Geographic Distribution of accD/bccp Alleles

**Figure 6:**
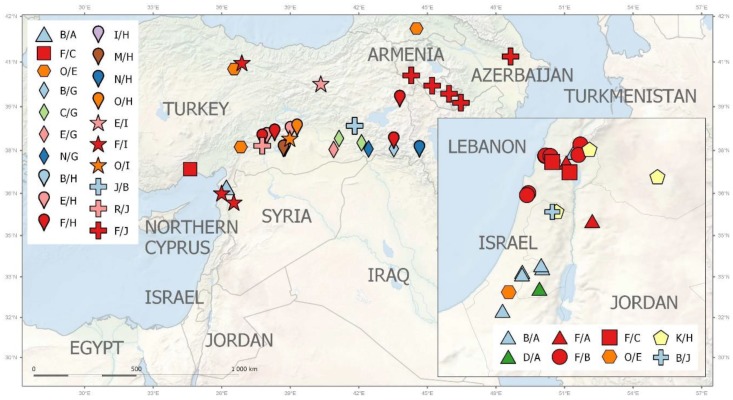
Geographic distribution of ACCD/BCCP allelic combinations assigned to large groups (for details see Table S1) within the Middle East.

*Pisum fulvum* (16 acc.) is geographically restricted to Israel (7 acc.), Syria (7 acc.), Jordan (1 acc.), and southeastern Turkey (1 acc.), and displays distinct *accD*/*bccp* alleles. Genetically and geographically the most diverse set is from *P. elatius* (96 acc.). Of these, there were 34 accessions from Turkey, which had the highest genetic diversity (Figure 6, Table S1). These accessions have various *accD*/*bccp* alleles, although the combination *accD*_25 and *bccp*_20 is the most frequent (10). The next large group is *P. elatius* from Israel, which had 25 accessions that belong to various genetic groups. These also have different *accD*_2 and *bccp*_5 (22 alleles occurring in 13 accessions). European samples cover a large region of Western (Spain, Portugal, France), Central (Italy), and Eastern (Greece, Hungary, Serbia) Europe (Table S1). The later samples are distinct by both by genome wide analyses and by *accD*/*bccp* alleles analysis. Finally, the most separate group of *P. elatius* is from Armenia, with unique *accD*_34 and *bccp*_21/22 alleles (Figure 6).

The cultivated pea is geographically less precisely localized, except for *P. abyssinicum*, which is found only in Ethiopia and Yemen. All *P. abyssinicum* accessions have *accD*_14/*bccp*_20/26 alleles. Landraces of *P. sativum* originate from 24 countries and span a large geographical area from the Western Mediterranean to Central and Southern Asia. They predominantly have *accD*_29 (41 acc.) and *bccp*_22 (36 acc.) alleles typical for cultivated pea. There are few distinct accessions that have different alleles. Two were from Algeria (*accD*_32/*bccp*_11/12), and two accessions were from Greece (specific *bccp*_3 allele). Two accessions from China (ATC6925, ATC6937) have a *accD*_6 allele shared with *P. elatius*, while PI560969 from Nepal has distinct *accD*_2/*bccp*_5 alleles (Table S1).

## 3. Discussion

Here we report the allelic composition and geographical distribution of two genes involved in postzygotic reproductive isolation in the pea [39]. Taking advantage of the available germplasm resources [52,53], we analyzed the allelic composition of chloroplast localized *accD* and nuclear encoded *bccp* genes. Our results extend the experimental data of Bogdanova et al. [39]. We analyzed the allelic composition of accessions collected from the wild (including all recognized *Pisum* species) and domesticated peas of various geographical origins.

Postzygotic reproductive isolation, expressed as hybrid sterility or inviability, hybrid weakness or necrosis, and hybrid breakdown, is considered one of the two major fundamental processes leading to speciation [2,9]. The plastome–genome dysfunctions concern various kinds of albinism. Generally, incompatible hybrid materials suffer from reduced pigment content, lower rates of photosynthesis, and an impaired thylakoid structure. We detected the occurrence of albinotic plants in crosses of wild *Pisum fulvum* or *P. elatius* with the cultivated pea *P. sativum*, which upon identification of the respective genes [39] prompted this study.

### 3.1. Hypervariability of the Chloroplast accD Gene

The region of the chloroplast genome around the *accD* gene has been found to be prone to accumulation of repeats, resulting in high interspecific variability in numerous species (*Pisum* and *Lathyrus* [45], *Capsicum* [54], *Glycine* [43], *Silene* [47], *Oenothera* [55,56], Cupressophytes [57]) but much less variability at the intraspecific level (*Medicago truncatula* [44], tea, *Camellia sinensis* [58], and pea, *Pisum sp*. [39,40]). Our present study substantially expands the previous reports [39,40] by analyzing 195 pea samples covering the entire geographical and species range [52,59]. Our results on the ratios of nonsynonymous to synonymous substitutions (*Ka*/*Ks*) in the pea *accD* gene agree with data from *Oenothera*, *Silene*, and Cupressophytes [47,55,57]. This indicates positive selection, since *Ka*/*Ks* values significantly above 1 are unlikely to occur without at least some of the mutations being advantageous. The large variation in plastid-encoded *accD* gene sequences, both between and within the *Pisum* species, is consistent with findings in *Silene*, where positive selection in the phylogenetic context has been detected [47]. In many cases of plastid genome evolution, mutations have disproportionately affected nonsynonymous sites, resulting in elevated ratios of nonsynonymous to synonymous substitution rates. Notably, plastid genome comparison between *Lathyrus sativus* and *Pisum sativum* resulted in identification of a region spanning the *accD* gene with increased mutation rate [45]. Analysis of publicly available *accD* sequences for *Lathyrus* and *Vicia* species supported these findings (unpublished).

Variation detected in the *Pisum* sp. *accD* sequence is mainly caused by the insertion of multiple tandem repeated sequences, as found in Cupressophytes [57] and *Medicago* [44]. In particular, the later study corresponds well to our pea *accD* data, since each of the 24 studied *Medicago truncatula* genotypes appears to have a different *accD* sequence, yet with maintained reading frames despite the high variability. Mapping of the insertion sites onto the predicted protein structure indicated their clustering within the N-terminal part of the ACCD protein that could not be reliably modelled due to intrinsic disorder. Such disordered protein regions are known to be extremely flexible and dynamic, alleviating some structural constraints [60], and were reported to be prone to insertions and deletions [61]. It has been suggested that regions surrounding tandem repeats evolve faster than other non-repeat-containing regions, which results in increased frequency of substitutions near the flanking sequences [62]. As shown in tobacco, a functional *accD* is essential for development [63]. Interestingly, the relationship to biparental inheritance of plastids was proposed to be related to the plastid competition [56]. Since about 20% of all angiosperms contain plastid DNA in the sperm cell, it is likely that this mechanism of cytonuclear conflict is also present in other systems [64,65,66,67].

### 3.2. Allelic accD/bccp Combinations Found in Wild and Domesticated Peas

One of our major aims was to detect allelic combinations of both genes occurring in wild peas, as well as in cultivated pea crop. Altogether we found 36 *accD* and 35 *bccp* alleles in the set of 195 accessions. Within the wild pea (*P. elatius*) these occurred in 60 out of 671 possible combinations, indicating a high diversity, while both domesticated *P. sativum* and *P. abyssinicum* had only a reduced subset. There was no overlap between *P. fulvum* and *P. elatius*, except for one *P. fulvum* JI2539 accession from Israel, which had *accD*_22 (G lineage) allele shared with three *P. elatius* samples from Turkey. Notably, in our previous study [52], we have found in this accession a typical *P. elatius trnSG_E6* allele, suggesting some past hybridization event between *P. fulvum* and *P. elatius*. Interestingly, in another two *P. fulvum* accessions (JI2510, JI2521) that also have the *trnSG_E6* allele [52], the *accD* allele was canonical to *P. fulvum* (*accD*_20, 21, e.g., F lineage). *P. abyssinicum* had *accD* alleles and combinations distinct from *P. sativum*, supporting its independent domestication [53]. The *accD*_14 allele of *P. abyssinicum* was not found in any of *P. elatius* or *P. sativum* samples. Notably, two of the most frequent alleles of each gene, *accD*_29 and *bccp*_22, contributed to the most frequent combination of *accD*_30/*bccp*_25 found in domesticated *P. sativum*.

It remains to be experimentally tested by crosses if the allelic combinations detected in the natural conditions create barriers against gene flow in natural pea populations. Some experimental crosses between cultivated pea and selected *P. fulvum* and *P. elatius* accessions were conducted by Bogdanova et al. [68]. These crosses revealed hybrid sterility, ultimately leading to identification of the respective genes [39]. In our work, we made reciprocal crosses between *P. elatius* L100 (*accD*_2/*bccp*_5) and *P. sativum* cv. Cameor (*accD*_29/*bccp*_22), which resulted in the appearance of albinotic plants (Smýkal, unpublished), while a cross between *P. elatius* JI64 (*accD*_30/*bccp*_5) and *P. sativum* JI92 (*accD*_29/*bccp*_22) was fully viable and fertile [69,70]. This corresponds to the findings of Bogdanova et al. (2015) [39] of a incompatible cross between *P. elatius* L100 (*accD*_2/*bccp*_5) and *P. sativum* WL12238 (*accD*_29/*bccp*_22); a cross between *P. elatius* JI1794 (*accD*_25/*bccp*_27), 721 (*accD*_5/*bccp*_22), and *P. abyssinicum* VIR 2759 (*accD*_14/*bccp*_26) were compatible with the cultivated pea *P. sativum* WL12238 (*accD*_29/*bccp*_22) [68]. Moreover, the existence of a second, unlinked, and yet unidentified nuclear *scs2* locus also involved in nuclear-cytoplasmic conflict has been proposed [39]. In this study, the authors proposed a model of determinants, based on seven substitutions and three deletions in ACCD and four amino acid substitutions in the biotinyl domain of BCCP protein. The results of our study add to this complexity, as there are far more possible combinations.

### 3.3. Domestication and Hybrid Incompatibility

In crops, artificial selection and hybridization accelerate the evolutionary process [71]. The majority of economically important crops were isolated from their progenitors through the existence of prezygotic or postzygotic reproductive barriers (or both), even though geographic isolation was absent during the domestication [38]. The reproductive barriers between wild crop progenitors and domesticated crops might be attributed to several mechanisms, including differences in karyotype or chromosomal rearrangements. Such karyotype differences are reported between *P. fulvum* and *P. elatius*, *P. sativum*, and between *P. sativum* and *P. abyssinicum* [72,73], and contribute to the partial fertility of the respective hybrids. Much less is known about the interactions between nuclear and cytoplasmic genomes. To date, only a few genes implicated in hybrid incompatibility have been isolated in crops. In maize, *Tcb1*, *Ga1*, and *Ga2* alleles influence interaction of pollen tubes with silk tissue and confer prezygotic barriers in crosses between cultivated *Zea mays* and the wild teosinte *Z. m. mexicana* [74]. About 50 loci controlling postzygotic reproductive barriers between rice subspecies have been identified and molecular products of some genes have been characterized [22]. For example, the *S5* locus, a determinant of *japonica-indica* sterility, is located in proximity to the domestication *OsC1* gene [75]. Similarly, the *Gn1a* gene involved in rice yield formation is linked with *S35*, which determines pollen sterility of *japonica-indica* hybrids [76]. Another example was shown in the tomato, where the *Cf-2* gene from wild *Lycopersicon pimpinellifolium* confers resistance to the fungus *Cladosporium fulvum* in an *Rcr3* dependent manner [48]; these two genes interact with each other to induce hybrid necrosis syndrome in the hybrids. Although the occurrence of albino plants in many interspecific crosses in crops is widely documented [77,78], its causes have not been studied in most cases. Notably, crosses between cultivated chickpea (*Cicer arietinum*) and its progenitor (*C. reticulatum*) yielded yellow and albino plants and a biparental plastid inheritance [77,78]. We speculate that this was caused by a similar mechanism as in the pea.

The results of this study might be relevant for breeding, particularly using more distant crop wild relatives, as well as hybrid crop breeding [79,80], but it remains to be tested by experimental crosses to identify causal effectors.

## 4. Material and Methods

### 4.1. Plant Material

We analyzed 195 previously described pea accessions (Smýkal et al. 2017, 2018, Trněný et al. 2018) [52,53,59], consisting of wild *P. elatius* (95) and *P. fulvum* (16) accessions (Table S1). Sixty domesticated *P. sativum* landraces and 24 domesticated *P. abyssinicum* accessions were selected to maximize the genetic diversity and to cover the entire range of the wild and landrace pea habitats. This span is approximately 5000 km in longitude from Morocco to Iran, and in latitude from Tunisia to Hungary; altitude ranged from sea level to about 2000 m. This material was previously morphologically described and assessed for its genetic diversity structure [52,53]. Plants were grown in 5 L pots with peat-sand (90:10) substrate mix (Florcom Profi, BB Com Ltd. Letohrad, Czech Republic), in glasshouse conditions (UP campus, Olomouc, Czech Republic).

### 4.2. DNA and RNA Analysis

Genomic DNA was isolated from a single plant per accession from approximately 100 mg of dry leaf material using the Invisorb Plant Genomic DNA Isolation kit (Invisorb, Berlin, Germany) and standard protocol [52,59]. Total RNA was isolated from young leaves using plant RNA kit (Macherey-Nagel, Düren, Germany). Isolated RNA was treated with DNaseI to remove genomic DNA. The *accD* gene was amplified directly from genomic DNA using primers (F1—GCATTAGTTTTCATTTTCAGTCC located 27 bp upstream of stop codon, R4—CTTTAATAGGGGTTTAGAATACA, located 94 bp upstream of ATG codon) [39]. We used cDNA as a template to avoid large intron sequences present in the *bccp*3 gene. One microgram of a total RNA was reversely transcribed with Oligo(dT) primer and AMV reverse transcriptase (Promega, Madison, USA) according to manufacturer´s protocol (Hradilová et al. 2017) [71]. Two step nested PCR amplification was used. After the first PCR (with primers F—CTAATGAAAGTGGCGGAAATC, R—CCTTATTACGCGTCTTAGTGAATG), the product was diluted (1:100) and the second PCR was performed (F33—CCATTCTCTGCACTCCCTTTCGCG, R1113—CAATTATTTCTCAATCTATTCAAAACG), using the conditions as described in Hradilová et al. [71]. PCR products were verified on a 1.5% agarose gel, treated with Exonuclease-Alkaline Phosphatase (Thermo Scientific, Brno, Czech Republic) and sequenced at Macrogene.

### 4.3. Sequence Analysis

For initial analysis, Geneious 7.1.7 (Biomatters Ltd., Auckland, New Zealand ) was used to edit and align sequences. Due to the presence of large gaps in the *accD* gene, sequences were translated into protein sequences, which reduced the overall length of the *accD* nucleotide alignment and partially helped to eliminate large gaps. This procedure reduced the complexity of the *accD* sequences. Sequences of the *bccp* gene were treated in the same manner, although these sequences were largely devoid of large indels. The translated protein sequences were aligned in Geneious using the MAFFT algorithm and the final alignment was manually adjusted. From the final alignment, different alleles and their frequencies were identified using the online tool FABOX [81].

To explore possible connections or relationships among the identified alleles, the reduced dataset (including each allele defined only once) was used for the network analysis. Several approaches of network construction were used (based on characters, Median network, Median-joining; based on distances, Neighbor network, Split decomposition) and implemented in SplitsTree [82]. The results were then compared. To compare the results of network analysis with a classically constructed bifurcating tree, a maximum parsimony (MP) tree was built using MEGA 6 with 1000 bootstrap replicates [83]. Because of the complex pattern of gaps within the *accD* gene, indels were treated as “partially deleted” (pairwise deletion, option implemented in MEGA) during the MP analysis. The final consensus tree was computed from all the equally parsimonious trees found during the analysis and was midpoint rooted. The tree topology was compared against the constructed networks. To simplify or reduce the number of identified alleles, groups of related alleles were inferred based on the constructed networks and the final consensus MP tree for both investigated genes. DnaSP v5.10 was used to determine nucleotide diversity and synonymous/non-synonymous sites ratios [84]. All studied *accD* and *bccp* sequences were deposited in the GenBank database under the accession numbers MK619486 to MK619678, and MK644626 to MK644819, respectively.

### 4.4. Tandem Repeat Analysis

Tandem repeats within DNA and protein sequences were identified in a combination of two algorithms (FastPCR [85] and RADAR [86]). The consensus DNA sequence of *accD* gene was first scanned by FastPCR at a repeat length ≥20 bp (k-mer = 12 with a tolerance for up to one mismatch within k-mer) with a similarity of above 70%. Potential tandem repeats for consensus protein sequence were further identified by RADAR software. Both methods complemented each other, since the boundaries of some degenerate and mixed tandem repeats were difficult to identify separately.

### 4.5. Protein Sequence Analysis and Structure Modelling

To identify the domains we used InterPro (www.ebi.ac.uk/interpro) and SMART databases (http://smart.embl-heidelberg.de). To generate molecular models of both proteins, standard sequences of the pea *accD* (GenBank YP_003587558.1) and *bccp* (GenBank DR89228.1) were used as queries to identify suitable templates and to perform molecular modelling by threading using Phyre2 in “normal” mode [87]. Only a partial model was generated for each protein, as portions of the sequence predicted to be disordered or lacking a suitable template (including some internal loops) could not be reliably modeled. In the case of ACCD, the structure of *Staphylococcus* acetyl-CoA carboxylase carboxyltransferase (PDB 2F9I) was identified as the best template. The second best template (PDB 2F9Y, also of bacterial origin) yielded a model of similar coverage and spatial organization. A similar model, also based on the PDB 2F9I template, was obtained for the same part of ACCD using another algorithm, RaptorX [88]. For BCCP, the best template identified by Phyre2 was the pyruvate carboxylase from *Methylobacillus flagellatus* (PDB 5KS8). The same template was also found by RaptorX as second best; namely, pyruvate carboxylase from *Listeria monocytogenes* (PDB 4QSH) yielded a spatially similar model. The Phyre2-generated models were subjected to additional refinement in the DeepView environment [89] to eliminate amino acid sidechain clashes. Subsequent evaluation of the resulting models using the WHAT_CHECK tools [90] revealed no critical errors, with scores for some parameters only slightly poorer than observed for the template for both proteins.

### 4.6. Mapping Protein Sequence Polymorphisms on Predicted Structure

Unique protein sequences encoded by alleles, each of the two loci were identified within aligned protein sequence sets using the ElimDupes tool at the Los Alamos HIV database website (https://www.hiv.lanl.gov/content/sequence/elimdupesv2/elimdupes.html). A map of polymorphisms was then generated manually from the resulting unique sequence alignments. A distribution of the polymorphisms between the modeled and non-modeled portions of the protein was statistically evaluated using the Chi-square test. 

## Figures and Tables

**Table 1:** Distribution of protein sequence polymorphisms in structurally modelled versus non-modelled parts of the ACCD and BCCP protein sequences.

Protein	Substitutions/Alignment Length		Indels/Alignment Length	
	Modelled	Not Modelled	Modelled	Not Modelled
***accD***	36/299	50/256*	2/299	17/256**
***bccp***	17/134	19/138	0/134	1/138

Asterisks denote significant differences in the frequency of the given category of mutations in non-modelled (disordered) parts of the protein compared to the modelled ones (*—*p* < 0.05, **—*p* < 0.01).

## Supplementary Materials

The following are available online at https://www.mdpi.com/1422-0067/20/7/1773/s1. Table S1: List and description of analyzed material, Table S2: Table of *accD*/*bccp* combinations, Figure S1: The alignment of amino acid sequences of all identified *accD* alleles, File S1: Theoretical model of pea BCCP protein structure, (co-ordinates in standard PDB format), File S2: Model of BCCP protein structure. Theoretical model of pea ACCD protein structure, (co-ordinates in standard PDB format). File S3: Theoretical model of pea BCCP protein structure, (co-ordinates in standard PDB format).
