# Supplementary material for: Allelic Diversity of Acetyl Coenzyme A Carboxylase accD/bccp Genes Implicated in Nuclear-Cytoplasmic Conflict in the Wild and Domesticated Pea (Pisum sp.)
Source: Int J Mol Sci. 2019 Apr 10;20(7):1773. doi: 10.3390/ijms20071773 (PMC6480052; doi:10.3390/ijms20071773)
Supplement: Supplementary file 1 [file ijms-20-01773-s001.zip › Figure S1.docx]

10 20 30 40 50 60 70 80 90 100 110 120 130 140 150 160 170

| | | | | | | | | | | | | | | | |

**Consensus** DSWKNNSENSSYSHADSLADVSNIDNLLSHKIFSIRDTNTNSNSNIYDIYYAY**DTNDTN**ITKYKWTNNINRCIESYLRSQICEDIDFNSDICDKVQSTIIILILILIRSTNDTN**DIS**DTN**DIS**ITKYKWTNNINRCIESYLRSQICEDIDFNSDICDKVQRTIIILIRSTNDTN**DIS**

**accD27** DSWKNNSENSSYSHADSLADVSNIDNLLSDKIFSIRD----SNSNIYDIYYAY**DTNDTN**ITKYKWTNNINRCIESYLRSQICEDIDFNSDICDKVQRTIIILILILIRSTNDTN**DISDTNDTN**ITKYKWTNNINRCIESYLRSQICEDIDFNSDICDKVQRTIIILIRSTNDTN**DIS**

**accD28** DSWKNNSENSSYSHADSLADVSNIDNLLSDKIFSIRD----SNSNIYDIYYAY**DTNDTN**ITKYKWTNNINRCIESYLRSQICEDIDFNSDICDKVQRTIIILI----RSTNDTN**DISDTNDTN**ITKYKWTNNINRCIESYLRSQICEDIDFNSDICDKVQRTIIILIRSTNDTN**DIS**

**accD30** DSWKNNSENSSYSHADSLADVSNIDNLLSHKIFSIRD----SNSNIYDIYYAY**DTNDTN**ITKYKWTNNINRCIESYLRSQICEDIDFNSDICDKVQRTIIILI----RSTNDTN**DIS**DTN**DIS**------------------------------------------------**DTNDTN**

**accD31** DSWKNNSENSSYSHADSLADVSNIDNLLSHKIFSIRD----SNSNIYDIYYAY**DTNDTN**ITKYKWTNNINRCIESYLRSQICEDIDFNSDICDKVQRTIIILI----RSTNDTN---DTN**DIS**------------------------------------------------DTN**DIS**

**accD33** DSWKNNSENSSYSHADSLADVSNIDNLLSHKIFSIRD----SNSNIYDIYYAY**DTNDTN**ITKYKWTNNINRCIESYLHSQICEDIDFNSDICDKVQRTIIILI----RSTNDTN---DTN**DIS**------------------------------------------------YTN**DIS**

**accD32** DSWKNNSENSSYSHADSLADVSNIDNLLSHKIFSIRD----SNSNIYDIYYAY**DTNDTN**ITKYKWTNNINRCIESYLRSQICEDIDFNSDICDKVQRTIIILI----RSTNDTN---DTN**DIS**------------------------------------------------DTN**DIS**

**accD22** DSWKNNSENSSYSHADSLADVSNIDNLLSHKIFSIRD----SNSNIYDIYYAY**DTNDTN**ITKYKWTNNINRCIASYLRSQICEDIDFNSDICDKVQRTIIILI----RSTNDTN**DIS**DTN**DIS**------------------------------------------------------

**accD24** DSWKNNSENSSYSHADSLADVSNIDNLLSHKIFSIRD----SNSNIYDIYYAY**DTNDTN**ITKYKWTNNINRCIESYLRSQICEDIDFNSDICDKVQRTIIILI----RSTNDTN**DIS**DTN**DIS**------------------------------------------------------

**accD25** DSWKNNSENSSYSHADSLADVSNIDNLLSHKIFSIRD----SNSNIYDIYYAY**DTNDTN**ITKYKWTNNINRCIESYLRSQICEDIDFNSDICDKVQRTIRILI----RSTNDTN**DIS**DTN**DIS**------------------------------------------------------

**accD34** DSWKNNSENSSYSHADSLADVSNIDNLLSHKIFSIRD----SNSNIYDIYYAY**DTNDTN**ITKYKWTNNINRCIESYLRSQICEDIDFNSDICDKVQRTIIILI----RSTNDTN**DIS**DTN**DIS**------------------------------------------------------

**accD26** DSWKNNSENSSYSHADSLADVSNIDNLLSDKIFSIRD----SNSNIYDIYYAY**DTNDTN**ITKYKWTNNINRCIESYLRSQICEDIDFNSDICDKVQRTIIILI----RSTNDTN**DIS**DTN**DIS**------------------------------------------------------

**accD29** DSWKNNSENSSYSHADSLADVSNIDNLLSDKIFSIRD----SNSNIYDIYYAY**DTNDTN**ITKYKWTNNINRCIESYLRSQICEDIDFNSDICDKVQRTIIILI----RSTNDTN**DIS**DTN**DIS**------------------------------------------------------

**accD30** DSWKNNSENSSYSHADSLADVSNIDNLLSDKIFSIRD----SNSNIYDIYYAY**DTNDTN**ITKYKWTNNINRCIESYLRSQICEDIDFNSDIFDKVQRTIIILI----RSTNDTN**DIS**DTN**DIS**------------------------------------------------------

**accD23** DSWKNNSENSSYSHADSLADVSNIDNLLSHKIFSIRD----SNSNIYDIYYAY**DTNDTN**ITKYKWTNNINRCIESYLRSQICEDIDFNSDICDKVQRTIIILI----RSTNDTN**DIS**DTN**DIS**------------------------------------------------------

**accD1** DSWKNNSENSSYSHADSLADVSNIDNLLSHTIFSIRD----SNSNIYDIYYAY**DTNDTN**ITKYKWTNNINRCIESYLRSQICEDIDFNSDICDKVQSTIIILI----RSTNDTN**DIS**DTN---------------------------------------------------------

**accD2** DSWKNNSENSSYSHADSLADVSNIDNLLSHTIFSIRD----SNSNIYDIYYAY**DTNDTN**ITKYKWTNNINRCIESYLRSQICEDIDFNSDICDKVQSTIIILI----RSTNDTN**DIS**DTN---------------------------------------------------------

**accD3** DSWKNNSENSSYSHADSLADVSNIDNLLSHKIFSIRD----SNSNIYDIYYAY**DTNDTN**ITKYKWTNNINRCIESYLRSQICEDIDFNSDICDKVQSTIIILI----RSTNDTN**DIS**DTN---------------------------------------------------------

**accD4** DSWKNNSENSSYSHADSLADVSNIDNLLSHKIFSIRD----SNSNIYDIYYAY**DTNDTN**ITKYKWTNNINRCIESYLRSQICEDIDFNSDICDKVQSTIIILI----RSTNDTN**DIS**DTN---------------------------------------------------------

**accD10** DSWKNNSENSSYSHADSLADVSNIDNLLSHKIFSIRD----SNSNIYDIYYAY**DTNDTN**ITKYKWTNNINRCIESYLRSQICEDIDFNSDICDKVQSTIIILI----RSTNDTN**DIS**DTN---------------------------------------------------------

**accD5** DSWKNNSENSSYSHADSLADVSNIDNLLSHKIFSIRD----SNSNIYDIYYAY**DTNDTN**ITKYKWTNNINRCIESYLRSQICEDIDFNSDICDKVQSTIIILI----TSTNDTN**DIS**DTN---------------------------------------------------------

**accD6** DSWKNNSENSSYSHADSLADVSNIDNLLSHKIFSIRD----SNSNIYDIYYAY**DTNDTN**ITKYKWTNNINRCIESYLRSQICEDIDFNSDICDKVQSTIIILI----TSTNDTN**DIS**DTN---------------------------------------------------------

**accD9** DSWKNNSENSSYSHADSLADVSNIDNLLSHKIFSIRD----SNSNIYDIYYAY**DTNDTN**ITKYKWTNNINRCIESYLRSQICEDIDFNSDICDKVQSTIIILI----RSTNDTN**DIS**DTN---------------------------------------------------------

**accD8** DSWKNNSENSSYSHADSLADVSNIDNLLSHKIFSIRD----SNSNIYDIYYAY**DTNDTN**ITKYKWTNNINRCIESYLRSQICEDIDFNSDICDKVQSTIIILI----RSTNDTN**DIS**DTN---------------------------------------------------------

**accD11** DSWKNNSENSSYSHADSLADVSNIDNLLSHKIFSIRD----SNSNIYDIYYAY**DTNDTN**ITKYKWTNNINRCIESYLRSQICEDIDFNSDICHKVQSTIIILI----RSTNDTN**DIS**DTN---------------------------------------------------------

**accD12** DSWKNNSENSSYSHADSLADVSNIDNLLSHKIFSIRD----SNSNIYDIYYAY**DTNDTN**ITKYKWTNNINRCIESYLRSQICEDIDFNSDICDKVQSTIIILI----RSTNDTN**DIS**DTN---------------------------------------------------------

**accD7** DSWKNNSENSSYSHADSLADVSNIDNLLSHKIFSIRD----SNSNIYDIYYAY**DTNDTN**ITKYKWTNNINRCIESYLRSQICEDIDFNSDICDKVQSTIIILI----RSTNDTN**DIS**DTN---------------------------------------------------------

**accD14** DSWKNNSENSSYSHADSLADVSNIDNLLSHKIFSIRD----SNSNIYDIYYAY**DTNDTN**ITKYKWTNNINRCIESYLRSQICEDIDFNSDICDKVQSTIIILI----RSTNDTN**DIS**DTN---------------------------------------------------------

**accD13** DSWKNNSENSSYSHADSLADVSNIDNLLSHKIFSIRD----SNSNIYDIYYAY**DTNDTN**ITKYKWTNNINRCIESYLRSQICEDIDFNSDICDKVQSTIIILI----RSTNDTN**DIS**DTN---------------------------------------------------------

**accD15** DSWKNNSENSSYSHADSLADVSNIDNLLSHKIFSIRD----SNSNIYDIDYAY**DTNDTN**ITKYKWTNNINRCIESYLRSQICEDIDFNSDICDKVQSTIIILI----RSTNDTN**DIS**DTN---------------------------------------------------------

**accD16** DSWKNNSENSSYSHADSLADVSNIDNLLSHKIFSIRD----SNSNIYDIDYAY**DTNDTN**ITKYKWTNNINRCIESYLRSQICEDIDFNSDICDKVQSTIIILI----RSTNDTN**DIS**DTN---------------------------------------------------------

**accD18** DSWKNNSENSSYNHADYLADVSNIDNLLSDKFFSIRNTN--SNSNIYDIYYAY**DTNDTN**----------------------------------------------------------------------------------------------------------------------

**accD21** DSWKNNSENSSYSHADYLADVSNIDNLLSDKFFSIRNTN--SNSNIYDIYYAY**DTNDTN**----------------------------------------------------------------------------------------------------------------------

**accD19** DSWKNNSENSSYRHADYLADVSNIDNLLSDKFFSIRNTN--SNSNIYDIYYAY**DTNDTN**----------------------------------------------------------------------------------------------------------------------

**accD20** DSWKNNSENSSYSHADYLADVSNIDNLLSDKFFSIRNTNTNSNSNIYDIYYAY**DTNDTN**----------------------------------------------------------------------------------------------------------------------

Zn-finger domain

**accD17** DSWKNNSENSSYKNADYLADVSNIDNLLSDKFFSIRN----SNSNIYDIYYAY**DTNDTN**----------------------------------------------------------------------------------------------------------------------

180 190 200 210 220 230 240 250 260 270 280 290 300 310 320 330 340 350

| | | | | | | | | | | | | | | | | |

**Consensus** DTNAIY**DISDTNDTN**DTNAIYDPF**DISDTNDTN**EIYDPFFILDINDTN**DISDTNDTN**DIYGIYDPDDIYETNIKDICERYSEIYRRNREKSTFVPIDYSDPNCMEKLARLWVQCETCYGLNFKQFFRPKMNICEHCGEHLKMSSSDRIDLSIDRDTWNPMDEDMVSVDPIKFDS

**accD27** DTN---**DISDTNDTN**DTNAIYDPF**DISDTNDTN**EIYDPFFILDIN------**DTNDTN**DIYGIYDPDDIYETNIKDICERYSEIYPRNREKSTFVPIDYSDPNCMEKLARLWVQCETCYGLNFKQFFRPKMNICEHCGEHLKMSSSDRIDLSIDRDTWNPMDEDMVSVDPIKFDS

**accD28** DTN---**DISDTNDTN**DTNAIYDPF**DISDTNDTN**EIYDPFFILDIN------**DTNDTN**DIYGIYDPDDIYETNIKDICERYSEIYPRNREKSTFVPIDYSDPNCMEKLARLWVQCETCYGLNFKQFFRPKMNICEHCGEHLKMSSSDRIDLSIDRDTWNPMDEDMVSVDPIKFDS

**accD30** DTNAIYDPF**DISDTNDTN**AIYDPF**DISDTNDTN**EIYDPFFILDIN------**DTNDTN**DIYGIYDPDDIYETNIKDICERYSEIYPRNREKSTFVPIDYSDPNCMEKLARLWVQCETCYGLNFKQFFRPKMNICEHCGEHLKMSSSDRIDLSIDRDTWNPMDEDMVSVDPIKFDS

**accD31** DTN------**DTNDTN**DTNAIYDPF**DISDTNDTN**EIYDPFFILDIN------**DTNDTN**DIYGIYDPDDIYETNIKDICERYSEIYPRNREKSTFVPIDYSDPNCMEKLARLWVQCETCYGLNFKQFFRPKMNICEHCGEHLKMSSSDRIDLSIDRDTWNPMDEDMVSVDPIKFDS

**accD33** DTN------**DTNDTN**DTNAIYDPF**DISDTNDTN**EIYDPFFILDIN------**DTNDTN**DIYGIYDPDDIYETNIKDICERYSEIYPRNREKSTFVPIDYSDPNCMEKLARLWVQCETCYGLNFKQFFRPKMNICEHCGEHLKMSSSDRIDLSIDRDTWNPMDEDMVSVDPIKFDS

**accD32** DTN------**DTNDTN**DTNAIYDPF**DISDTNDTN**EIYDPFFILDIN------**DTNDTN**DIYGIYDPDDIYETNIKDICERYSEIYPRNREKSTFVPIDYSDPNCMEKLARLWVQCETCYGLNFKQFFRPKMNICEHCGEHLKMSSSDRIDLSIDRDTWNPMDEDMVSVDPIKFDS

**accD22** DTN------**DTNDTN**DTNAIYDPF**DISDTNDTN**EIYDPFFILDIN------**DTNDTN**DIYGIYDPDDIYETNIKDICERYSEIYPRNREKSTFVPIDYSDPNCMEKLARLWVECETCYGLNFKPFFRPKMNICEHCGEHLKMSSSDRIDLSIDRDTWNPMDEDMVSVDPIKFDS

**accD24** DTN------**DTNDTN**DTNAIYDPF**DISDTNDTN**EIYDPFFILDIN------**DTNDTN**DIYGIYDPDDIYETNIKDICERYSEIYPRNREKSTFVPIDYSDPNCMEKLARLWVQCETCYGLNFKPFFRPKMNICEHCGEHLKMSSSDRIDLSIDRDTWNPMDEDMVSVDPIKFDS

**accD25** DTN------**DTNDTN**DTNAIYDPF**DISDTNDTN**EIYDPFFILDIN------**DTNDTN**DIYGIYDPDDIYETNIKDICERYSEIYPRNREKSTFVPIDYSDPNCMEKLARLWVQCETCYGLNFKPFFRPKMNICEHCGEHLKMSSSDRIDLSIDRDTWNPMDEDMVSVDPIKFDS

**accD34** ---------**DTNDTNDTN**AIYDPF**DISDTNDTN**EIYDPFFILDIN------**DTNDTN**DIYGIYDPDDIYETNIKDICERYSEIYPRNREKSTFVPIDYSDPNCMEKLARLWVQCETCYGLNFKQFFRPKMNICEHCGEHLKMSSSDRIDLSIDRDTWNPMDEDMVSVDPIKFDS

**accD26** ---------**DTNDTNDTN**AIYDPF**DISDTNDTN**EIYDPFFILDIN------**DTNDTN**DIYGIYDPDDIYETNIKDICERYSEIYPRNREKSTFVPIDYSDPNCMEKLARLWVQCETCYGLNFKQFFRPKMNICEHCGEHLKMSSSDRIDLSIDRDTWNPMDEDMVSVDPIKFDS

**accD29** ---------**DTNDTNDTN**AIYDPF**DISDTNDTN**EIYDPFFILDIN------**DTNDTN**DIYGIYDPDDIYETNIKDICERYSEIYPRNREKSTFVPIDYSDPNCMEKLARLWVQCETCYGLNFKQFFRPKMNICEHCGEHLKMSSSDRIDLSIDRDTWNPMDEDMVSVDPIKFDS

**accD30** ---------**DTNDTNDTN**AIYDPF**DISDTNDTN**EIYDPFFILDIN------**DTNDTN**DIYGIYDPDDIYETNIKDICERYSEIYPRNREKSTFVPIDYSDPNCMEKLARLWVQCETCYGLNFKQFFRPKMNICEHCGEHLKMSSSDRIDLSIDRDTWNPMDEDMVSVDPIKFDS

**accD23** ------**DTNDTNDTNDTN**AIYDPF**DISDTNDTN**EIYDPFFILDIN------**DTNDTN**DIYGIYDPDDIYETNIKDICERYSEIYPRNREKSTFVPIDYSDPNCMEKLARLWVECETCYGLNFKPFFRPKMNICEHCGEHLKMSSSDRIDLSIDRDTWNPMDEDMVSVDPIKFDS

**accD1** ------------**DTNDTN**AIYDPF**DISDTNDTN**EIYDPFFILDINDTN**DISDTNDTN**DIYGIYDPDDIYE--------RYSEIYRRNR-KSTFVPIDYIHPNCMEKLARLWVQCRTCYGLNFKPFFRPKMNICEHCGEHLKMSSSDRIDLSIDRDTWNPMDEDMVSVDPIKFDS

**accD2** ------------**DTNDTN**AIYDPF**DISDTNDTN**EIYDPFFILDINDTN**DISDTNDTN**DIYGIYDPDDIYE--------RYSEIYRRNR-KSTFVPIDYIHPNCMEKLARLWVQCRTCYGLNFKPFFRPKMNICEHCGEHLKMSSSDRIDLSIDRDTWNPMDEDMVSVDPIKFDS

**accD3** ------------**DTNDTN**AIYDPF**DISDTNDTN**EIYDPFFILDINDTN**DISDTNDTN**DIYGIYDPDDIYETNIKDICERYSEIYRRNREKSTFVPIDYSDPNCMEKLARLWVQCETCYGLNFKQFFRPKMNICEHCGEHLKMSSSDRIDLSIDRDTWNPMDEDMVSVDPIKFDS

**accD4** ------------**DTNDTN**AIYDPF**DISDTNDTN**EIYDPFFILDINDTN**DISDTNDTN**DIYGIYDPDDIYETNIKDICERYSEIYRRNREKSTFVPIDYSDPNCMEKLARLWVQCETCYGLNFKQFFRPKMNICEHCGEHLKMSSSDRIDLSIDRDTWNPMDEDMVSVDPIKFDS

**accD10** ------------**DTNDTN**AIYDPF**DISDTNDTN**EIYDPFFILDINDTN**DISDTNDTN**DIYGIYDPDDIYETNIKDICERYSEIYRRNREKSTFVPIDYSDPNSMEKLARLWVQCETCYGLNFKQFFRPKMNICEHCGEHLKMSSSDRIDLSIDRDTWNPMDEDMVSVDPIKFDS

**accD5** ------------**DTNDTN**AIYDPF**DISDTNDTN**EIYDPFFILDINDTN**DISDTNDTN**DIYGIYDPDDIYETNIKDICERYSEIYRRNREKSTFVPIDYSDPNCMEKLARLWVQCETCYGLNFKQFFRPKMNICEHCGEHLKMSSSDRIDLSIDRDTWNPMDEDMVSVDPIKFDS

**accD6** ------------**DTNDTN**AIYDPF**DISDTNDTN**EIYDPFFILDINDTN**DISDTNDTN**DIYGIYDPDDIYETNIKDICERYSEIYRRNREKSTFVPIDYSDPNCMEKLARLWVQCETCYGLNFKQFFRPKMNICEHCGEHLKMSSSDRIDLSIDRDTWNPMDEDMVSVDPIKFDS

**accD9** ------------**DTNDTN**AIYDPF**DISDTNDTN**EIYDPFFILDINDTN**DISDTNDTN**DIYGIYDPDDIYETNIKDICERYSEIYRRNREKSTFVPIDYSDPNSMEKLARLWVQCETCYGLNFKQFFRPKMNICEHCGEHLKMSSSDRIDLSIDRDTWNPMDEDMVSVDPIKFDS

**accD8** ------------**DTNDTN**AIYDPF**DISDTNDTN**EIYDPFFILDINDTN**DISDTNDTN**DIYGIYDPDDIYETNIKDICERYSEIYRRNREKSTFVPIDYSDPNSMEKLARLWVQCKTCYGLNFKQFFRPKMNICEHCGEHLKMSSSDRIDLSIDRDTWNPMDEDMVSVDPIKFDS

**accD11** ------------**DTNDTN**AIYDPF**DISDTNDTN**EIYDPFFILDINDTN**DISDTNDTN**DIYGIYDPDDIYETNIKDICERYSEIYRRNREKSTFVPIDYSDPNSMEKLARLWVQCETCYGLNFKQFFRPKMNICEHCGEHLKMSSSDRIDLSIDRDTWNPMDEDMVSVDPIKFDS

**accD12** ------------**DTNDTN**AIYDPF**DISDTNDTN**EIYDPFFILDINDTN**DISDTNDTN**DIYGIYDPDDIYETNIKDICERYSEIYRRNREKSTFVPIDYSDPNSMEKLARLWVQCETCYGLNFKQFFRPKMNICEHCGEHLKMSSSDRIDLSIDRDTWNPMDEDMVSVDPIKFDS

**accD7** ------**DISDTNDTN**DTNAIYDPF**DISDTNDTN**EIYDPFFILDINDTN**DISDTNDTN**DIYGIYDPDDIYETNIKDICERYSEIYRRNREKSTFVPIDYSDPNCMEKLARLWVQCETCYGLNFKQFFRPKMNICEHCGEHLKMSSSDRIDLSIDRDTWNPMDEDMVSVDPIKFDS

**accD14** ------**DISDTNDTN**DTNAIYDPF**DISDTNDTN**EIYDPFFILDINDTN**DISDTNDTN**DIYGIYDPDDIYETNIKDICERYNEIYRRNREKSTFVSIDYSDPNCMEKLARLWVQCETCYGLNFKQFFRPKMNICEHCGEHLKMSSSDRIDLSIDRDTWNPMDEDMVSVDPIKFDS

**accD13** ------------**DTNDTN**AIYDPF**DISDTNDTN**EIYDPFFILDINDTN**DISDTNDTN**DIYGIYDPDDIYETNIKDICERYNEIYRRNREKSTFVSIDYSDPNCMEKLARLWVQCETCYGLNFKQFFRPKMNICEHCGEHLKMSSSDRIDLSIDRDTWNPMDEDMVSVDPIKFDS

**accD15** ------------**DTNDTN**AIYDPF**DISDTNDTN**EIYDPFFILDIN---------DTNDIYGIYDRDDIYETNIKDICERYSEIYPRNREKRTFVPIDYSDPNCMEKLARLWVQCETCYGLNFKQFFRPKMNICEHCGEHLKMSSSDRIDLSIDRDTWNPIDEDMVSVDPIKFDS

**accD16** ------------**DTNDTN**AIYDPF**DISDTNDTN**EIYDPFFILDIN---------DTNDIYGIYDRDDIYETNIKDICERYSEIYPRNREKRTFVPIDYSDPNCMEKLARLWVQCETCYGLNFKQFFRPKMNICEHCGEHLKMSSSDRIDLSIDRDTWNPMDEDMVSVDPIKFDS

**accD18** ---------------DTNAIYDPFDILDINDTN---------------**DISDTNDTN**DIYGIYDRDDIYETNIKHIWERYSEIYRRNREKSTFVTIDYSDPNCMEKLARLWVQCKTCYGLNFQQFFRPKMNICEHCGEHLKMSSSDRIDLSIDRDTWNPMDEDMVSLDPIQFDS

**accD21** ---------------DTNAIYDPFDILDINDTN---------------**DISDTNDTN**DIYGIYDRDDIYETNIKHIWERYSEIYRRNREKSTFVTIDYSDPNCMEKLARLWVQCKTCYGLNFQQFFRPKMNICEHCGEHLKMSSSDRIDLSIDRDTWNPMDEDMVSLDPIQFDS

**accD19** ---------------DTNAIYDPFDILDINDTN---------------**DISDTNDTN**DIYGIYDRDDIYETNIKHIWERYSEIYRRNREKSTFVTIDYSDPNCMEKLARLWVQCKTCYGLNFQQFFRPKMNICEHCGEHLKMSSSDRIDLSIDRDTWNPMDEDMVSLDPIQFDS

**accD20** ---------------DTNAIYDPFDILDINDTN---------------**DISDTNDTN**DIYGIYDRDDIYETNIKHIWERYSEIYRRNREKSTFVTIDYSDPNCMEKLARLWVQCKTCYGLNFQQFFRPKMNICEHCGEHLKMSSSDRIDLSIDRDTWNPMDEDMVSLDPIQFDS

**accD17** ---------------DTNAIYDPFDILDINDTN---------------**DISDTNDTN**DIYGIYDRDDIYETNIKHIWERYSEIYRRNREKSTFVTIDYSDPNCMEKLARLWVQCKTCYGLNFQQFFRPKMNICEHCGEHLKMSSSDRIDLSIDRDTWNPMDEDMVSLDPIQFDS

acetyl-CoA- binding site coA-carboxylation catalytic

360 370 380 390 400 410 420 430 440 450 460 470 480 490 500 510 520

| | | | | | | | | | | | | | | | |

**Consensus** GIKELGSEEESSKDRLDEDMLSPDPIELDSEEESSKDRVDSEEEKDQSYIDRLDSYQEKTGLPETVQTGTDQREEINRLFEDIMNQLDLYLYLQTAKNRVDSEEEEEEKDQSYIDRLDSYQEKTGLPEAVQTGTGQLNGPILALAVMDSEEFIAGSMGCVVGEKITRLIE

**accD27** -IKELGSEEESSKDRLDEDMLSPDPIELDSEEESSKDRVDSEEEKDQSYIDRLDSYQEKTGLPETVQTGTDQREEIHPLFEDIMNQLDLYL--QTAKNRVDSEEE---KDQSYIDRLDSYQEKTGLPEAVQTGTGQLNGPILALAVMDSEEFIAGSMGCVVGEKITRLIE

**accD28** -IKELGSEEESSKDRLDEDMLSPDPIELDSEEESSKDRVDSEEEKDQSYIDRLDSYQEKTGLPETVQTGTDQREEIHPLFEDIMNQLDLYL--QTAKNRVDSEEE---KDQSYIDRLDSYQEKTGLPEAVQTGTGQLNGPILALAVMDSEEFIAGSMGCVVGEKITRLIE

**accD30** -IKELGSEEESSKDRLDEDMLSPDPIELDSEEESSKDRVDSEEEKDQSYIDRLDSYQEKTGLPETVQTGTDQREEIHPLFEDIMNQLDLYL--QTAKNRVDSEEE---KDQSYIDRLDSYQEKTGLPEAVQTGTGQLNGPILALAVMDSEEFIAGSMGCVVGEKITRLIE

**accD31** -IKELGSEEESSKDRLDEDMLSPDPIELDSEEESSKDRVDSEEEKDQSYIDRLDSYQEKTGLPETVQTGTDQREEIHPLFEDIMNQLDLYL--QTAKNRVDSEEE---KDQSYIDRLDSYQEKTGLPEAVQTGTGQLNGPILALAVMDSEEFIAGSMGCVVGEKITRLIE

**accD33** -IKELGSEEESSKDRLDEDMLSPDPIELDSEEESSKDRVDSEEEKDQSYIDRLDSYQEKTGLPETVQTGTDQREEIHPLFEDIMNQLDLYL--QTAKNRVDSEEE---KDQSYIDRLDSYQEKTGLPEAVQTGTGQLNGPILALAVMDSEEFIAGSMGCVVGEKITRLIE

**accD32** GIKELGSEEESSKDRLDEDMLSPDPIELDSEEESSKDREDSEEEKDQSYIDRLDSYQEKTGLPETVQTGTDQREEIHPLFEDIMNQLDLYL--QTAKNRVDSEEE---KDQSYIDRLDSYQEKTGLPEAVQTGTGQLNGPILALAVMDSEEFIAGSMGCVVGEKITRLIE

**accD22** -IKELGSEEESSKDRLDEDMLSPDPIELDSEEESSKDRVDSEEEKDQSYIDRLDSYQEKTGLPETVQTGTDQREEIHPLFEDIMNQLDLYL--QTAKNRVDSEEE---KDQSYIDRLDSYQEKTGLPEAVQTGTGQLNGPILALAVMDSEEFIAGSMGCVVGEKITRLIE

**accD24** -IKELGSEEESSKDRLDEDMLSPDPIELDSEEESSKDRVDSEEEKDQSYIDRLYSYQEKTGLPETVQTGTDQREEIHPLFEDIMNQLDLYL--QTAKNRVDSEEE---KDQSYIDRLDSYQEKTGLPEAVQTGTGQLNGPILALAVMDSEEFIAGSMGCVVGEKITRLIE

**accD25** -IKELGSEEESSKDRLDEDMLSPDPIELDSEEESSKDRVDSEEEKDQSYIDRLDSYQEKTGLPETVQTGTDQREEIHPLFEDIMNQLDLYL--QTAKNRVDSEEE---KDQSYIDRLDSYQEKTGLPEAVQTGTGQLNGPILALAVMDSEEFIAGSMGCVVGEKITRLIE

**accD34** -IKELGSEEESSKDRLDEDMLSPDPIELDSEEESSKDRVDSEEEKDQSYIDRLDSYQEKTGLPETVQTGTDQREEIHPLFEDIMNQLDLYL--QTAKNRVDSEEE---KDQSYIDRLDSYQEKTGLPEAVQTGTGQLNGPILALAVMDSEEFIAGSMGCVVGEKITRLIE

**accD26** -IKELGSEEESSKDRLDEDMLSPDPIELDSEKESSKDRVDSEEEKDQSYIDRLDSYQEKTGLPETVQTGTDQREEIHPLFEDIMNQLDLYL--QTAKNRVDSEEE---KDQSYIDRLDSYQEKTGLPEAVQTGTGQLNGPILALAVMDSEEFIAGSMGCVVGEKITRLIE

**accD29** -IKELGSEEESSKDRLDEDMLSPDPIELDSEEESSKDRVDSEEEKDQSYIDRLDSYQEKTGLPETVQTGTDQREEIHPLFEDIMNQLDLYL--QTAKNRVDSEEE---KDQSYIDRLDSYQEKTGLPEAVQTGTGQLNGPILALAVMDSEEFIAGSMGCVVGEKITRLIE

**accD30** -IKELGSEEESSKDRLDEDMLSPDPIELDSEEESSKDRVDSEEEKDQSYIDRLDSYQEKTGLPETVQTGTDQREEIHPLFEDIMNQLDLYL--QTAKNRVDSEEE---KDQSYIDRLDSYQEKTGLPEAVQTGTGQLNGPILALAVMDSEEFIAGSMGCVVGEKITRLIE

**accD23** -IKELGSEEESSKDRLD-----------------------SEEEKDQSYIDRLDSYQEKTGLPETVQTGTDQREEIHPLFEDIMNQLDLYL--QTATNRVDSEEE---KDQSYIDRLDSYQEKTGLPEAVQTGTGQLNGPILALAVMDSEEFIAGSMGCVVGEKITRLIE

**accD1** -IKELGSEEE----------------------------------KDQSYIDRLDSYQEKTGLPETVQTGTDQREEINRLFQDIMKKLDLYLYLQTAKNRVDSEEEEEEKDPSYIDRLDSYQEKTGLPEAVQTGTGQLNGPILALAVMDSEEFIAGSMGCVVGEKITRLIE

**accD2** -IKELGSEEE----------------------------------KDQSYIDRLDSYQEKTGLPETVQTGTDQREEINRLFQDIMKKLDLYL--QTAKNRVDSEEEE--KDPSYIDRLDSYQEKTGLPEAVQTGTGQLNGPILALAVMDSEEFIAGSMGCVVGEKITRLIE

**accD3** -IKELGSEEE----------------------------------KDQSYIDRLDSYQEKTGLPETVQTGTDQREEINRLFQDIMKKLDLYLYLQTAKNRVDSEEEEEEKDPSYIDRLDSYQEKTGLPEAVQTGTGQLNGPILALAVMDSEEFIAGSMGCVVGEKITRLIE

**accD4** -IKELGSEEE----------------------------------KDQSYIDRLDSYQEKTGLPETVQTGTDQREEINRLFEDIMNKLDLYL--QTAKNRVDSEEEEEEKDPSYIDRLDSYQEKTGLPEAVQTGTGQLNGPILALAVMDSEEFIAGSMGCVVGEKITRLIE

**accD10** -IKELGSEEE----------------------------------KDQSYIDRLDSYQEKTGLPQTVQTGTDQREEINRLFEDIMNKLDLYL--QTAKNRVDSEEEEEEKDQSYIDRLDSYQEKTGLPEAVQTGTGQLNGPILALAVMDSEEFIAGSMGCVVGEKITRLIE

**accD5** -IKELGSEEE-------------------------------EEEKDPSYIDRLDSYQEKTGLPETVQTGTDQREEINRLFEDIMNKLDLYL--QTAKNRVDSEEEEEEKDPSYIDRLDSYQEKTGLPEAVQTGTGQLNGPILALAVMDSEEFIAGSMGCVVGEKITRLIE

**accD6** -IKELGSEEE-------------------------------EEEKDQSYIDRLDSYQEKTGLPETVQTGTDQREEINRLFEDIMNKLDLYL--QTAKNRVDSEEEEEEKDPSYIDRLDSYQEKTGLPEAVQTGTGQLNGPILALAVMDSEEFIAGSMGCVVGEKITRLIE

**accD9** -IKELGSEEE-------------------------------EEEKDQSYIDRLDSYQEKTGLPETVQTGTDQREEINRLFEDIMNKLDLYL--QTAKNRVDSEEEEEEKDQSYIDRLDSYQEKTGLPEAVQTGTGQLNGPILALAVMDSEEFIAGSMGCVVGEKITRLIE

**accD8** -IKELGSEEE-------------------------------EEEKDQSYIDRLDSYQEKTGLPETVQTGTDQREEINRLFEDIMNKLDLYL--QTAKNRVDSEEEEEEKDQSYIDRLDSYQEKTGLPEAVQTGTGQLNGPILALAVMDSEEFIAGSMGCVVGEKITRLIE

**accD11** -IKELGSEEE-------------------------------EEAKDQSYIDRLDSYQEKTGLPETVQTGTDQREEINRLFEDIMNKLDLYL--QTAKNRVDSEEEEEAKDQSYIDRLDSYQEKTGLPEAVQTGTGQLNGPILALAVMDSEEFIAGSMGCVVGEKITRLIE

**accD12** -IKELGSEEE-------------------------------EEEKDQSYIDRLDSYQEKTGLPETVQTGTDQREEINRLFEDIMNKLDLYL--QTAKNRVDSEEEE--KDQSYIDRLDSYQEKTGLPEAVQTGTGQLNGPILALAVMDSEEFIAGSMGCVVGEKITRLIE

**accD7** -IKELGSEEE----------------------------------KDQSYIDRLDSYQEKTGLPETVQTGTDQREEINRLFEDIMNKLDLYL--QTAKNRVDSEEEEEEKDPSYIDRLDSYQEKTGLPEAVQTGTGQLNGPILALAVMDSEEFIAGSMGCVVGEKITRLIE

**accD14** -IKELGSEEE----------------------------------KDQSYIDRLDSYQEKTGLPETVQTGTAQREESNRLFEDIMNKLDLYL--QTAKNRVDSEEE---KDQSYIDRLDSYQEKTGLPEAVQTGTGQLNGPILALAVMDSEEFIAGSMGCVVGEKITRLIE

**accD13** -IKELGSEEE----------------------------------KDQSYIDRLDSYQEKTGLPETVQTGTAQREESNRLFEDIMNKLDLYL--QTAKNRVDSEEE---KDQSYIDRLDSYQEKTGLPEAVQTGTGQLNGPILALAVMDSEEFIAGSMGCVVGEKITRLIE

**accD15** -IKELGSEEE----------------------------------KDKSYIDRLDSYQEKTGLPETVQTGTDQREEINRLFEDIMNKLDLYL--QTAKNRVDSEEE---KDQSYMDRLDSYQEKTGLPEAVQTGTGQLNGPILALAVMDSEEFIAGSMGCVVGEKITRLIE

**accD16** -IKELGSEEE----------------------------------KDQSYIDRLDSYQEKTGLPETVQTGTDQREEINRLFEDIMNKLDLYL--QTAKNRVDSEEE---KDQSYMDRLDSYQEKTGLPEAVQTGTGQLNGPILALAVMDSEEFIAGSMGCVVGEKITRLIE

**accD18** -IKELSSEDESSKDRVDS-------------EEKKEKKKLSYMDRLDRYIERLDSYQKKKGLPETVQTGTDQRKEINRLFEDIMNQLDLSL--QTAKNRVYSEEE---KDASYMDRLDSYQEITGLPEAVQTGTGQLNGPILALAVMDSEEFIAGSMGCVVGEKITRLIE

**accD21** -IKELSSEDESSKDRVDS-------------EEKKEKKKLSYMDRLDRYIERLDSYQKKKGLPETVQTGTDQRKEINRLFEDIMNQLDLSL--QTAKNRVYSEEE---KDASYMDRLDSYQEITGLPEAVQTGTGQLNGPILALAVMDSEEFIAGSMGCVVGEKITRLIE

**accD19** -IKELSSEDESSKDRVDS-------------EEKKEKKKLSYMDRLDRYIERLDSYQKKKGLPETVQTGTDQRKEINRLFEDIMNQLDLSL--QTAKNRVYSEEE---KDASYMDRLDSYQEITGLPEAVQTGTGQLNGPILALAVMDSEEFIAGSMGCVVGEKITRLIE

**accD20** -IKELSSEDESSKDRVDS-------------EEKKEKKKLSYMDRLDRYIERLDSYQKKKGLPETVQTGTDQRKEINRLFEDIMNQLDLSL--QTAKNRVYSEEE---KDASYMDRLDSYQEITGLPEAVQTGTGQLNGPILALAVMDSEEFIAGSMGCVVGEKITRLIE

**accD17** -IKELSSEDESSKDRVDS-------------EEKKEKKKLSYMDRLDRYIERLDSYQKKKGLPETVQTGTDQRKEINRLFEDIMNQLDLSL--QTAKNRVYSEEE---KDASYMDRLDSYQEITGLPEAVQTGTGQLNGPILALAVMDSEEFIAGSMGCVVGEKITRLIE

carboxybiotin binding site

530 540 550 560 570 580 590 600 610 620 630

| | | | | | | | | | |

**Consensus** YATNLLLPLIIVCASGGARMQEGSLSLMQMAKISSALYNYQINQKLFYVAILTSPTTGGVTASFGMLGDIIIAEPNATIAFAGKRVIEQLLNKEVPEGSQSADLLFDRGLLDAVVPRH

**accD27** YATNLLLPLIIVCASGGARMQEGSLSLMQMAKISSALYNYQINQKLFYVAILTSPTTGGVTASFGMLGDIIIAEPNATIAFAGKRVIEQLLNKEVPEGSQSADLLFDRGLLDAVVPRH

**accD28** YATNLLLPLIIVCASGGARMQEGSLSLMQMAKISSALYNYQINQKLFYVAILTSPTTGGVTASFGMLGDIIIAEPNATIAFAGKRVIEQLLNKEVPEGSQSADLLFDRGLLDAVVPRH

**accD30** YATNLLLPLIIVCASGGARMQEGSLSLMQMAKISSALYNYQINQKLFYVAILTSPTTGGVTASFGMLGDIIIAEPNATIAFAGKRVIEQLLNKEVPEGSQSADLLFDRGLLDAVVPRH

**accD31** YATNLLLPLIIVCASGGARMQEGSLSLMQMAKISSALYNYQINQKLFYVAILTSPTTGGVTASFGMLGDIIIAEPNATIAFAGKRVIEQLLNKEVPEGSQSADLLFDRGLLDAVVPRH

**accD33** YATNLLLPLIIVCASGGARMQEGSLSLMQMAKISSALYNYQINQKLFYVAILTSPTTGGVTASFGMLGDIIIAEPNATIAFAGKRVIEQLLNKEVPEGSQSADLLFDRGLLDAVVPRH

**accD32** YATNLLLPLIIVCASGGARMQEGSLSLMQMAKISSALYNYQINQKLFYVAILTSPTTGGVTASFGMLGDIIIAEPNATIAFAGKRVIEQLLNKEVPEGSQSADLLFDRGLLDAVVPRH

**accD22** YATNLLLPLIIVCASGGARMQEGSLSLMQMAKISSALYNYQINQKLFYVVILTSPTTGGVTASFGMLGDIIIAEPNATIAFAGKRVIEQLLNKEVPEGSQSADLLFDRGLLDAVVPRH

**accD24** YATNLLLPLIIVCASGGARMQEGSLSLMQMAKISSALYNYQINQKLFYVAILTSPTTGGVTASFGMLGDIIIAEPNATIAFAGKRVIEQLLNKEVPEGSQSADLLFDRGLLDAVVPRH

**accD25** YATNLLLPLIIVCASGGARMQEGSLSLMQMAKISSALYNYQINQKLFYVAILTSPTTGGVTASFGMLGDIIIAEPNATIAFAGKRVIEQLLNKEVPEGSQSADLLFDRGLLDAVVPRH

**accD34** YATNLLLPLIIVCASGGARMQEGSLSLMQMAKISSALYNYQINQKLFYVAILTSPTTGGVTASFGMLGDIIIAEPNATIAFAGKRVIEQLLNKEVPEGSQSADLLFDRGLLDAVVPRH

**accD26** YATNLLLPLIIVCASGGARMQEGSLSLMQMAKISSALYNYQINQKLFYVAILTSPTTGGVTASFGMLGDIIIAEPNATIAFAGKRVIEQLLNKEVPEGSQSADLLFDRGLLDAVVPRH

**accD29** YATNLLLPLIIVCASGGARMQEGSLSLMQMAKISSALYNYQINQKLFYVAILTSPTTGGVTASFGMLGDIIIAEPNATIAFAGKRVIEQLLNKEVPEGSQSADLLFDRGLLDAVVPRH

**accD30** YATNLLLPLIIVCASGGARMQEGSLSLMQMAKISSALYNYQINQKLFYVAILTSPTTGGVTASFGMLGDIIIAEPNATIAFAGKRVIEQLLNKEVPEGSQSADLLFDRGLLDAVVPRH

**accD23** YATNLLLPLIIVCASGGARMQEGSLSLMQMAKISSALYNYQINQKLFYVAILTSPTTGGVTASFGMLGDIIIAEPNATIAFAGKRVIEQLLNKEVPEGSQSADLLFDRGLLDAVVPRH

**accD1** YATNLLLPLIIVCASGGARMQEGSLSLMQMAKISSALYNYQINQKLFYVAILTSPTTGGVTASFGMLGDIIIAEPNATIAFAGKRVIEQLLNKQVPEGSQSADLLFDRGLLDAVVPRH

**accD2** YATNLLLPLIIVCASGGARMQEGSLSLMQMAKISSALYNYQINQKLFYVAILTSPTTGGVTASFGMLGDIIIAEPNATIAFAGKRVIEQLLNKQVPEGSQSADLLFDRGLLDAVVPRH

**accD3** YATNLLLPLIIVCASGGARMQEGSLSLMQMAKISSALYNYQINQKLFYVAILTSPTTGGVTASFGMLGDIIIAEPNATIAFAGKRVIEQLLNKQVPEGSQSADLLFDRGLLDAVVPRH

**accD4** YATNLLLPLIIVCASGGARMQEGSLSLMQMAKISSALYNYQINQKLFYVAILTSPTTGGVTASFGMLGDIIIAEPNATIAFAGKRVIEQLLNKEVPEGSQSADLLFDRGLLDAVVPRH

**accD10** YATNLLLPLIIVCASGGARMQEGSLSLMQMAKISSALYNYQINQKLFYVAILTSPTTGGVTASFGMLGDIIIAEPNATIAFAGKRVIEQLLNKEVPEGSQSADLLFDRGLLDAVVPRH

**accD5** YATNLLLPLIIVCASGGARMQEGSLSLMQMAKISSALYNYQINQKLFYVAILTSPTTGGVTASFGMLGDIIIAEPNATIAFAGKRVIEQLLNKEVPEGSQSADLLFDRGLLDAVVPRH

**accD6** YATNLLLPLIIVCASGGARMQEGSLSLMQMAKISSALYNYQINQKLFYVAILTSPTTGGVTASFGMLGDIIIAEPNATIAFAGKRVIEQLLNKEVPEGSQSADLLFDRGLLDAVVPRH

**accD9** YATNLLLPLIIVCASGGARMQEGSLSLMQMAKISSALYNYQINQKLFYVAILTSPTTGGVTASFGMLGDIIIAEPNATIAFAGKRVIEQLLNKEVPEGSQSADLLFDRGLLDAVVPRH

**accD8** YATNLLLPLIIVCASGGARMQEGSLSLMQMAKISSALYNYQINQKLFYVAILTSPTTGGVTASFGMLGDIIIAEPNATIAFAGKRVIEQLLNKEVPEGSQSADLLFDRGLLDAVVPRH

**accD11** YATNLLLPLIIVCASGGARMQEGSLSLMQMAKISSALYNYQINQKLFYVAILTSPTTGGVTASFGMLGDIIIAEPNATIAFAGKRVIEQLLNKEVPEGSQSADLLFDRGLLDAVVPRH

**accD12** YATNLLLPLIIVCASGGARMQEGSLSLMQMAKISSALYNYQINQKLFYVAILTSPTTGGVTASFGMLGDIIIAEPNATIAFAGKRVIEQLLNKEVPEGSQSADLLFDRGLLDAVVPRH

**accD7** YATNLLLPLIIVCASGGARMQEGSLSLMQMAKISSALYNYQINQKLFYVAILTSPTTGGVTASFGMLGDIIIAEPNATIAFAGKRVIEQLLNKEVPEGSQSADLLFDRGLLDAVVPRH

**accD14** YATNLLLPLIIVCASGGARMQEGSLSLMQMAKISSALYNYQINQKLFYVAILTSPTTGGVTASFGMLGDIIIAEPNATIAFAGKRVIEQLLNKEVPEGSQSADLLFDRGLLDAVVPRH

**accD13** YATNLLLPLIIVCASGGARMQEGSLSLMQMAKISSALYNYQINQKLFYVAILTSPTTGGVTASFGMLGDIIIAEPNATIAFAGKRVIEQLLNKEVPEGSQSADLLFDRGLLDAVVPRH

**accD15** YATNRLLPLIIVCASGGARMQEGSLSLMQMAKISSALYNYQINQKLFYVAILTSPTTGGVTASFGMLGDIIIAEPNATIAFAGKRVIEQLLNKQVPEGSQSADLLFDKGLLDAVVPRH

**accD16** YATNRLLPLIIVCASGGARMQEGSLSLMQMAKISSALYNYQINQKLFYVAILTSPTTGGVTASFGMLGDIIIAEPNATIAFAGKRVIEQLLNKQVPEGSQSADLLFDKGLLDAVVPRH

**accD18** YATNLLLPLIIVCASGGARIQEGSLSLMQMAKISSALYNYQINKKLFYVAILTSPTAGGVTASFGMLGDIIIAEPNATIAFAGKRVIEQLLNKEVPEGSQSADLLFDKGLLDAVVPRH

**accD21** YATNLLLPLIIVCASGGARIQEGSLSLMQMAKISSALYNYQINKKLFYVAILTSPTAGGVTASFGMLGDIIIAEPNATIAFAGKRVIEQLLNKEVPEGSQSADLLFDKGLLDAVVPRH

**accD19** YATNLLLPLIIVCASGGARIQEGSLSLMQMAKISSALYNYQINKKLFYVAILTSPTAGGVTASFGMLGDIIIAEPNATIAFAGKRVIEQLLNKEVPEGSQSADLLFDKGLLDAVVPRH

**accD20** YATNLLLPLIIVCASGGARIQEGSLSLMQMAKISSALYNYQINKKLFYVAILTSPTAGGVTASFGMLGDIIIAEPNATIAFAGKRVIEQLLNKEVPEGSQSADLLFDKGLLDAVVPRH

**accD17** YATNLLLPLIIVCASGGARIQEGNLSLMQMAKISSALYNYQINKKLFYVAILTSPTAGGVTASFGMLGDIIIAEPNATIAFAGKRVIEQLLNKEVPEGSQSADLLFDKGLLDAVVPRH
